# Exploring the antineoplastic potential of α-mangostin in breast cancer

**DOI:** 10.1007/s13659-025-00528-5

**Published:** 2025-07-03

**Authors:** Daniela Amador-Martínez, Mizraim Flores, Rafael Vargas-Castro, Rocío García-Becerra, Euclides Avila, Lorenza Díaz, Janice García-Quiroz

**Affiliations:** 1 Departamento de Biología de La Reproducción Dr. Carlos Gual Castro, Instituto Nacional de Ciencias Médicas y Nutrición Salvador Zubirán, Av. Vasco de Quiroga No. 15, Belisario Domínguez Sección XVI, Tlalpan, C.P. 14080 Ciudad de Mexico, México; 2 Departamento de Biología Molecular y Biotecnología, Instituto de Investigaciones Biomédicas, Universidad Nacional Autónoma de México, Av. Universidad 3000, Coyoacán, C.P. 04510 Ciudad de Mexico, México

**Keywords:** α-Mangostin, Breast cancer, Antineoplastic effects, Garcinia mangostana, Xanthone

## Abstract

Among women, breast cancer is the most frequently diagnosed cancer and the leading cause of cancer-related mortality globally. Despite improvements in early detection and diagnosis, some risk factors have been on the rise, including the decline in birth rate, the use of oral contraceptives, and the escalation in alcohol consumption and obesity. Thus, there is an imperative urgent need to expand accessible prevention and treatment options for breast cancer. Regarding these tumors, several natural compounds have shown efficacy in slowing or preventing their progression, offering a promising therapeutic alternative. Among these, α-mangostin, a xanthone derived from mangosteen, has demonstrated promising antitumor effects against different malignancies, particularly breast cancer. The mechanisms involved in α-mangostin´s therapeutic effects include downregulation of oncogenic ion channels, modulation of cell cycle progression, suppression of oncogene expression, and interference with steroid and growth factor receptors signaling. This review thoroughly explores these mechanisms, as well as updates information on α-mangostin chemical structure and its potential as a coadjuvant to conventional breast cancer therapies. Furthermore, we provide scientifically supported insights for the development of clinically applicable α-mangostin-based treatments, highlighting the robust body of evidence supporting its cancer-fighting properties, despite the absence of clinical studies to date.

**Graphical Abstract:**

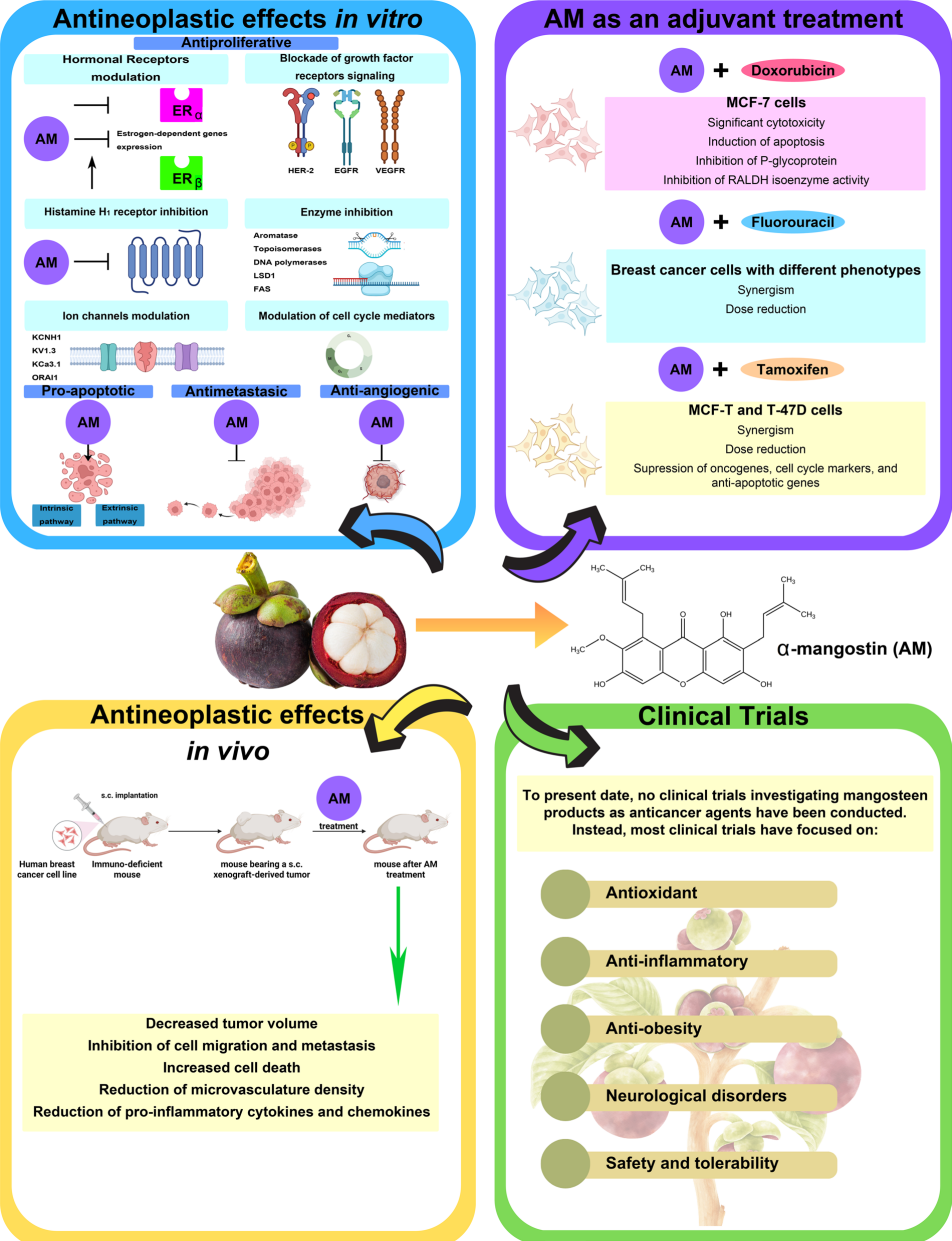

**Supplementary Information:**

The online version contains supplementary material available at 10.1007/s13659-025-00528-5.

## Introduction

As estimated by GLOBOCAN 2022, breast cancer represents the most frequently diagnosed cancer type among women and the leading cause of cancer-related mortality worldwide, which accounts for one in six cancer deaths [1]. Moreover, mortality rates caused by this malignancy are higher in less developed countries, posing a heavy burden for healthcare systems and representing a clinical concern worldwide [1]. This underscores the urgent need for accessible therapeutic strategies with low toxicity, alongside early detection and timely diagnosis. Plant-derived bioactive molecules have been extensively studied for their therapeutic potential, with the mangosteen tree (*Garcinia mangostana*) emerging as a notable source of antineoplastic phytochemicals, particularly xanthones. Among these, α-mangostin has gained significant attention due to its ability to inhibit the proliferation of various breast cancer cell types in in vitro studies and xenografted murine models. In these preclinical studies, α-mangostin has demonstrated the potential to enhance the efficacy of conventional cancer therapies, such as doxorubicin, 5-fluorouracil, and tamoxifen, by allowing dose reductions while improving therapeutic outcomes [2–4]. This combination of low cost, reduced toxicity, and diverse antineoplastic mechanisms makes α-mangostin a promising candidate for developing novel breast cancer treatments. Notably, α-mangostin, exhibits significant anticancer properties across various cancer types, as reviewed elsewhere [5–8]. Beyond its antineoplastic effects, α-mangostin offers additional health benefits, including antibacterial, antifungal, and antiparasitic activities, as well as antioxidant, antidiabetic, antiobesity, and antiallergic effects. These attributes position α-mangostin as a valuable natural health product for improving overall health and supporting the treatment of various medical conditions [5].

This review provides an updated overview of the anticancer effects of α-mangostin, particularly in breast cancer, emphasizing its cellular and molecular mechanisms. The antineoplastic effects of α-mangostin include modulation of breast hormonal receptors, inhibition of growth factor and histamine H_1_ receptor (H_1_R) signaling, and targeting key enzymes and cell cycle mediators. α-Mangostin promotes apoptosis and inhibits cancer cell adhesion, invasion, and metastasis through mechanisms such as epithelial-mesenchymal transition (EMT) and focal adhesion kinase (FAK) inhibition. Additionally, α-mangostin exhibits antiangiogenic and immunomodulatory effects. Preclinical studies and clinical trials supporting its potential as an adjuvant therapy are also discussed.

### Taxonomy of mangosteen

**Fig. 1:**
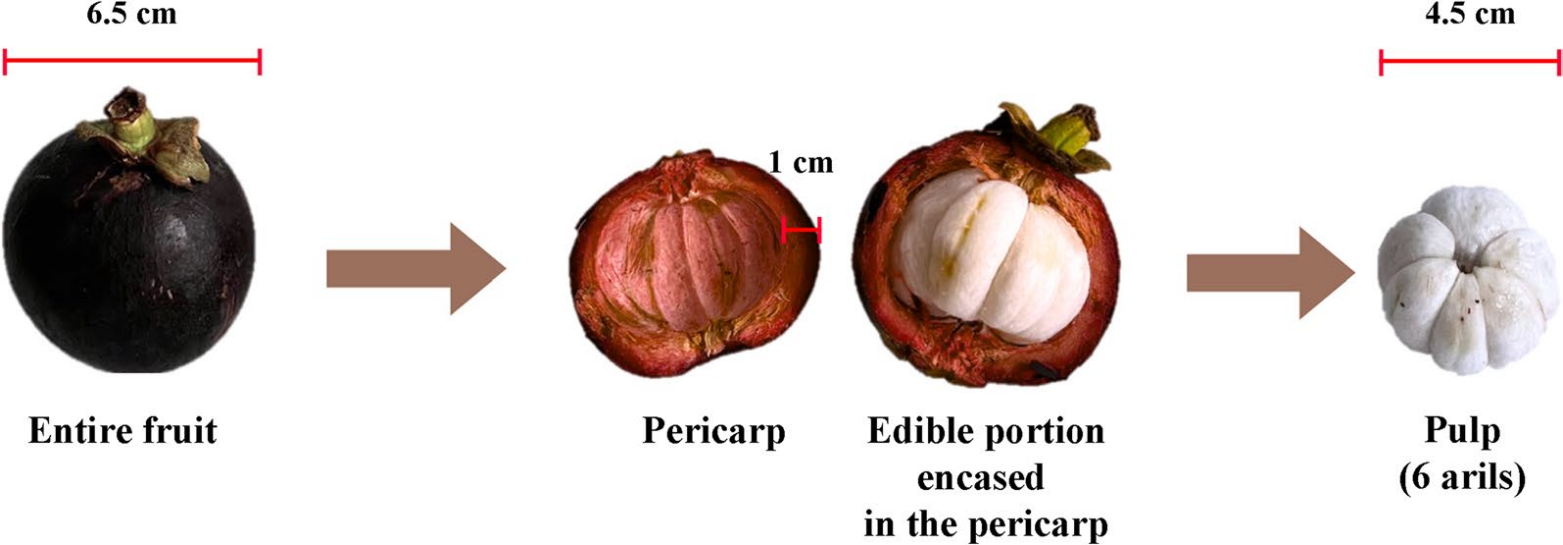
Mangosteen fruit. The mangosteen fruit has a thick outer rind, known as the pericarp (rind or peel), which measures approximately 1.0 cm in thickness and encases the inner white pulp. The pulp, about 4.5 cm in diameter, consists of 4 to 7 soft, juicy arils with a sweet yet slightly acidic flavor. The entire fruit has an average diameter of 6.5 cm

The mangosteen tree (*Garcinia mangostana*) belongs to the *Garcinia* genus within the *Clusiaceae* family, commonly known as *Guttiferae*. It can grow up to 25 m, featuring a central trunk, symmetrical branches, and dense foliage. Its leaves measure 15–25 cm in length and 4.5–13 cm in width. The flowers have four petals, measuring 2–5 cm long, and are either yellow-green with red edges or predominantly red [9–11]. These flowers produce mangosteen fruits, which are encased in a thick, hard, deep-red pericarp that encloses 4 to 7 arils (Fig. 1). Mangosteen trees grow slowly, requiring 6 to 9 years for the first harvest [9, 10].

The mangosteen tree, native from Southeast Asia, is extensively cultivated in Malaysia, Indonesia, Thailand, the Philippines, and certain regions of India and Sri Lanka. It has also been introduced to tropical regions in Australia, Africa, and America. Particularly in this last Continent, the mangosteen tree can be found in South and Central America, including Mexico [10, 12, 13].

### Traditional medicinal applications of mangosteen and its fruit-derived supplements

Globally, mangosteen has a long history of use in traditional medicine, particularly in Southeast Asia. Various parts of the tree, including the leaves, bark, roots, heartwood, seeds, pericarp, and fruit, have been utilized in different forms, such as powders, ointments, tinctures, and teas to treat numerous medical conditions. As an antimicrobial agent, mangosteen has been employed to treat bacterial infections and parasitic diseases, including dysentery and cholera [14]. It also addresses other gastrointestinal issues like aphthae, abdominal pain, hemorrhoids, and diarrhea, and is considered a digestive aid. Externally, it treats skin infections, wounds, chronic ulcers, and suppurations [12, 15]. Additionally, mangosteen is used to treat genito-urinary diseases such as urethra suppuration, cystitis, leucorrhea, gonorrhea, vaginal thrush, and menstrual disorders, as well as respiratory diseases like tuberculosis [12, 15]. Its potent anti-inflammatory properties are effective for arthritis, skin disorders such as acne, eczema, hyperkeratosis, and psoriasis, as well as allergic reactions [12, 16]. In addition to traditional uses, mangosteen fruit, pericarp and whole fruit have been incorporated into a variety of dietary supplements, including capsules, tablets, powders, and beverages (Supplementary Table 1). However, it is important to note that these formulations have not been tested in clinical studies as anticancer agents.

Mangosteen is rich in bioactive compounds, including xanthones, terpenes, anthocyanins, and tannins. Xanthones are the predominant bioactive secondary metabolites, responsible for the most significant biological activities attributed to this fruit. Xanthones have been identified in different parts of the plant; however, most are concentrated in the pericarp of mangosteen fruit [12, 15].

## Xanthones from *Garcinia mangostana*

**Fig. 2:**
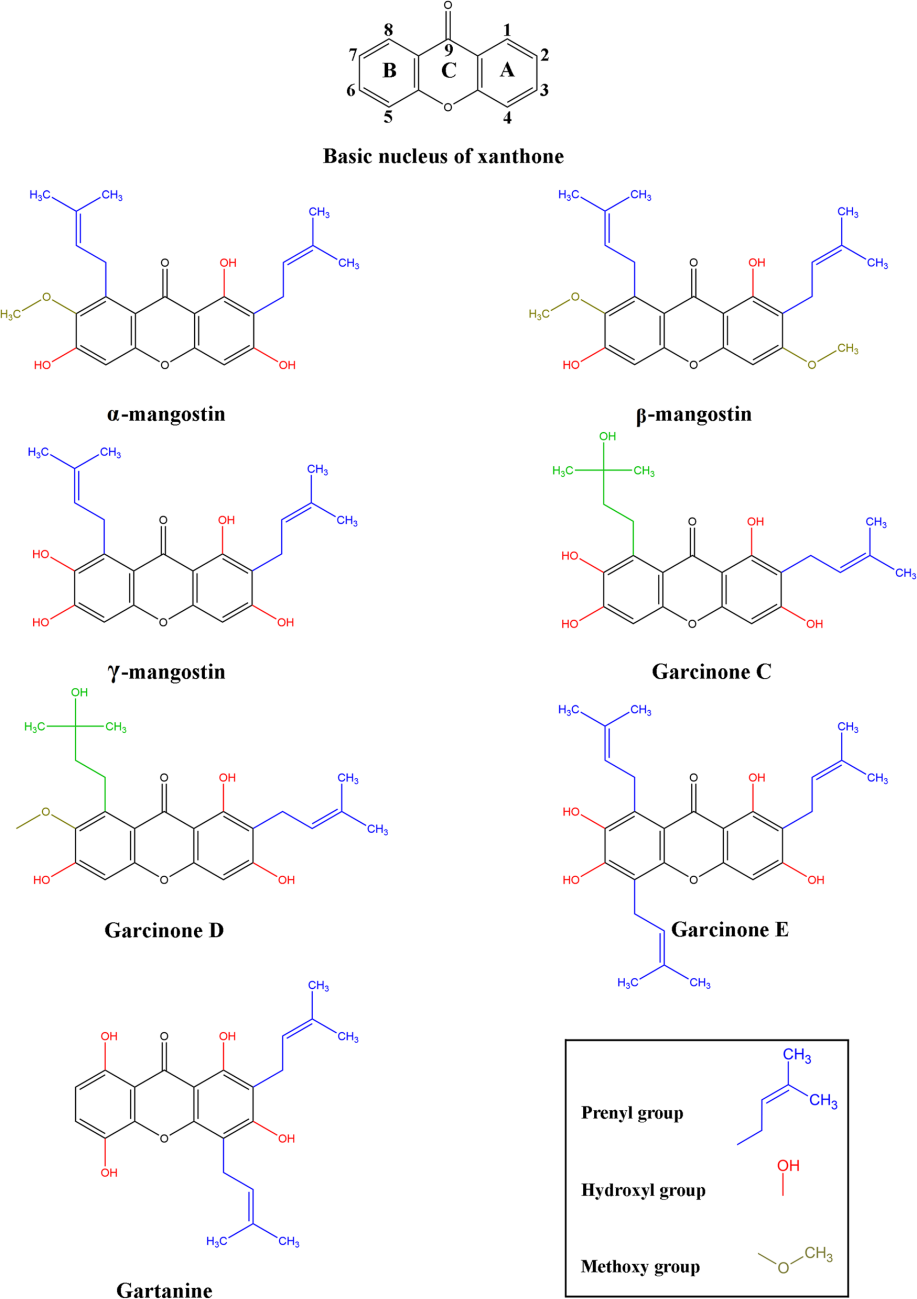
Chemical structure of the major prenylated xanthones from mangosteen pericarp. The xanthone nucleus is an aromatic oxygenated heterocyclic molecule with a planar, symmetric, and tricyclic structure, comprising two aromatics rings: A-ring (carbons 1–4) and B-ring (carbons 5–8), connected by the C ring (γ-pyrone ring, carbon 9). The prenylated xanthones feature prenyl groups and vary in the number and position of these groups, as well as the hydroxyl and methoxy groups attached to the xanthone nucleus. These variations are believed to significantly influence the biological activities of the xanthones. The structures were created in ChemDraw version 20.0.0.41 (1998–2000 PerkinElmer informatics, Inc)

The pericarp exudes a yellow resin, hence the term “xanthones”, since the word xanthone is derived from the Greek word *xanthós*, meaning yellow. Xanthone is an aromatic oxygenated heterocyclic molecule with a planar, symmetric and tricyclic structure named dibenzo-γ-pyrone or 9H-xanthen-9-one. Xanthone has the molecular formula C_13_H_8_O_2_, its structure includes two aromatic rings: A-ring and B-ring, connected by γ-pyrone ring (C-ring), as shown in Fig. 2 [17].

The xanthone nucleus is considered a “privileged structure” due to its ability to interact with various biological targets. This capability arises from its planar and rigid heteroaromatic tricyclic ring system that can accommodate a diverse group of substituents around these rings, the central carbonyl group capable of multiple interactions, and the biaryl ether group (two aromatic rings connected by an oxygen atom) contributing to the electronic system [18]. The xanthone nucleus can be substituted with various functional groups through processes such as alkylation, hydroxylation, prenylation, alkoxylation, and acylation, among others. Prenylated xanthones are the major group of naturally occurring xanthones, recognized for their anti-proliferative effects, attributed to the presence of prenyl groups (chains of carbon atoms derived from isoprene units) at key positions on the xanthone nucleus (Fig. 2) [18]. These prenyl groups facilitate the internalization of the molecule and influence protein interactions [15].

Structure–activity relationship studies indicate that both prenyl and hydroxyl groups on the xanthone nucleus are essential for promoting anti-proliferative effects [19]. By comparing the cytotoxicity of different xanthones and analyzing their structures, it has been concluded that the hydroxyl group at the C1 position and prenyl substituents at the C2 position in the xanthone nucleus are crucial for its cytotoxic effects [19, 20]. Hydroxylation at the C3 position enhances cytotoxicity, while its replacement by the methoxy group decreases the potency to cause mitochondrial dysfunction [15]. The cytotoxicity of xanthones is generally reduced by the hydroxylation of the prenylated chain in the C8, as observed in garcinone C and D, compared to α-mangostin and γ-mangostin [20]. The synthesis of α-mangostin derivatives highlights the significance of hydroxyl groups on C3 and C6 for cytotoxic activity. Di-substitution of these groups diminishes cytotoxicity against cancer cell lines, emphasizing the importance of retaining these hydroxyl groups. Additionally, the hydroxyl group at C7 plays a crucial role, replacing it with a methoxy group decreases cytotoxicity. Therefore, the number and locations of hydroxyl groups have varied effects on cytotoxicity, whereas oxidation of the prenyl group at C2 has limited impact. Moreover, Structure–activity relationship studies determined that maintaining the prenyl group at C8 is essential for retaining the cytotoxicity of xanthones; however, oxidizing the prenyl group at C8 results in the loss of this cytotoxicity [21, 22].

Interestingly, it was determined that certain xanthones could inhibit the cyclin-dependent kinase-4 (CDK4) protein, requiring specific functional groups for that, such as isoprenyl groups at the C2 and C8 positions, with hydroxyl groups extending from 3 and 7 positions, as well as the lack of isoprenyl group at the C4 position. The inhibition of CDK4 prevents the phosphorylation of downstream targets, which is crucial for stopping uncontrolled cell cycle progression. Vemu et al*.* hypothesized that the isoprenyl group at the C2 position of α-mangostin can bind deep within the ATP-binding pocket of CDK4. However, when the isoprenyl group is at C4, and absent at C8, as in gartanine, there is no inhibition of CDK4. It is possible that an isoprenyl group at C4 obstructs the binding of the xanthone to the CDK4 ATP-binding domain [22]. Hydroxylation of key groups is critical for inhibiting CDK4. Among α-mangostin, β-mangostin, and γ-mangostin, hydroxylation at the C3 and C7 positions resulted in the most potent inhibition, with γ-mangostin being the most effective. However, the in vitro assay did not consider phase I metabolism, which may occur in vivo. It is possible that methoxy groups, such as those in α-mangostin (C7) and β-mangostin (C3 and C7), might undergo O-demethylation to generate γ-mangostin in vivo. When comparing γ-mangostin to garcinone D, hydroxylation of the isoprenyl group appeared to counteract the methoxy group at C7, thereby restoring CDK4 inhibition [22].

The most abundant mangostins in mangosteen fruit are α-mangostin, followed by γ-mangostin and β-mangostin. Other notable xanthones include garcinone E, gartanin, and 8-deoxygartanin. Their relative abundance varies depending on the extraction method used, as shown in Table 1.

**Table 1:** Major xanthones extracted from mangosteen fruit tissues

Xanthone	Pericarp, rind or peel (mg/kg)						Arils, pulp and seeds (mg/kg)			
α-Mangostin	13,650	11,300	256,000	11,733	55,100	25,400	1,000	400	~ 37	276
γ-Mangostin	2,600	1,200		3,036		4,200	100	82		
β-Mangostin		30	9,000		1,700					
Gartanin	340	1,400	14,000	704	2,100			55		
8-Deoxy gartanin	30		20,000	500	15,000			50		
Garcinone E	30		14,000	484				93		
Extraction	Pericarp macerated in methanol, dichloromethane-extracted	Pericarp benzene extraction	Powdered rinds extracted with supercritical CO_2_ extraction	Pericarps extracted with dichloro-methane, redissolved in methanol	Grounded rinds extracted with 80:20 acetone–water solution	Powdered pericarps extracted with ethyl acetate	Fruit pulp powdered, then extracted with ethyl acetate	Arils extracted with dichloro-methane and reconstituted in methanol	Arils were acetone-extracted, and ethyl acetate-fractionated	Arils and seeds extracted with methanol
Ref	[23]	[24]	[25]	[26]	[27]	[28]	[28]	[26]	[24]	[29]

Xanthones were purified using column chromatography with various solvents. The extracted xanthones quantities were adjusted to mg *per* kg of fruit tissue weight

The α-mangostin was the first xanthone isolated from the mangosteen pericarp, identified by W. Schmid in 1855. Between 1930 and 1932, Dragendorf and Murakami elucidated its structure, followed by Yates and Stout, who established its molecular formula in 1958 [12].

## Therapeutic benefits of α-mangostin: antineoplastic properties

The α-mangostin has a wide range of medicinal properties, including antioxidant [23], antineoplastic [30], anti-inflammatory [31, 32], antihistamine [33], antibacterial [34], antifungal [35], antiviral [36–44], antimalarial [45], antidiabetic [46], antihyperlipidemic [46], cardioprotective [47, 48], hepatoprotective [49], neuroprotective [50], and immunomodulatory activities [51], among others.

In terms of its antineoplastic properties, α-mangostin has shown chemopreventive and antitumoral effects against various cancers, including cholangiocarcinoma [52], pheochromocytoma [53], glioblastoma [54], osteosarcoma [55], head and neck cancer [56], prostate cancer [57, 58], gastric cancer [59], pancreatic cancer [60–62], lung cancer [63], cervical cancer [43, 64], colon cancer [65], ovarian cancer [66, 67], skin cancer [68], renal cancer [69], and breast cancer [3, 70–79].

### Mechanisms of α-mangostin involved in inhibiting breast cancer cell proliferation

In the context of breast cancer, while personalized therapeutic strategies have been developed and applied based on specific tumor phenotypes, α-mangostin has shown antiproliferative effects regardless of the tumor molecular expression profile. This includes its effectiveness in breast cancer cells representing tumor phenotypes such as Estrogen Receptor-positive (ER +), Human Epidermal Growth Factor Receptor Type 2 (HER-2) enriched, and Triple Negative (TN), which lacks expression of ER, Progesterone Receptor (PR), and HER-2 (Table 2).

**Table 2:** Antiproliferative effects of α-mangostin or mangosteen extract on breast cancer cell lines

Breast cancer cell line (phenotype)	Concentration and time of treatment	Assay	Results	Ref
MCF-7 (ER +)	0, 2, 4, 6, 8, 10, 12, 14, and 16 µM/24 and 48 h	MTT	α-Mangostin inhibited cell proliferation in a time- and concentration-dependent manner	[79]
	MRE (0–500 µg/mL for 24 h)	MTT	The MRE reduced cell viability with an IC_50_ = 167.23 µg/mL	[80]
	The cells were treated with increasing concentrations of α-mangostin for 72 h	SRB	α-Mangostin inhibited cell proliferation with an IC_50_ value of 51.5 ± 2.8 µM	[81]
	The cells were treated with serial concentrations of α-mangostin for 48 h	MTT	α-Mangostin induced cytotoxic activity with an IC_50_ value of 10.81 ± 1.12 µM	[21]
MCF-7 (ER +) MCF-7-CR (ER +)	0–30 μM/12 h, 24 h, and 48 h	Alamar blue or MTT	α-Mangostin induced loss of cell viability in both a time- and concentration-dependent manner. The IC_50_ values were 30.83 ± 1.85 µM for MCF-7 cells and 28.72 ± 1.98 µM for MCF-7 CR at 12 h; 3.29 ± 1.02 µM for MCF-7 cells, and 2.53 ± 1.36 µM for MCF-7 CR at 24 h; and 1.17 ± 0.53 µM for MCF-7 cells, and 1.05 ± 0.73 µM for MCF-7-CR at 48 h	[82]
	30 µM/1–5 days		α-Mangostin-induced time-dependent loss of cell viability of MCF-7 spheroid cells	
MCF-7 (ER +) T-47D (ER +)	0–10 µM/6 days	SRB	α-Mangostin significantly inhibited MCF-7 and T-47D cell proliferation in a concentration-dependent manner, with an IC_50_ of 3.53 ± 0.23 and 7.15 ± 0.16, respectively	[4]
MCF-7 (ER +) MDA-MB-231 (TN)	0–10 µM/24 and 48 h	CCK-8	α-Mangostin exhibits strong cytotoxic effects in vitro against MDA-MB-231 and MCF-7 cells with IC_50_ = 3.35 µM and 3.57 µM respectively at 24 h and IC_50_ = 2.60 µM and 2.74 µM at 48 h	[75]
MCF-7 (ER +) MDA-MB-231 (TN)	1, 5 and 10 µM/48 h	SRB	α-Mangostin inhibited MCF-7 cell proliferation in a concentration-dependent manner, whereas MDA-MB-231 cells were less sensitive. The authors suggest that xanthone inhibits growth through the ER. Additionally, suppression of the ER and the estrogen-dependent gene Ps2 was observed in MCF-7 cells treated with 10 µM of α-mangostin. Knockdown of ER levels with siRNA attenuated the effects of α-mangostin	[76]
MCF-7 (ER +) MDA-MB-231 (TN) MCF-10A (normal mammary epithelial cells)	0.5–5 μM/24 h	Resazurin	α-Mangostin significantly reduced cell viability in a concentration-dependent manner, with an IC_50_ = 4 μM, and 2.5 μM for MCF-7 and MDA-MB-231, respectively; reassuring that α-mangostin suppresses selectively the cell viability of breast cancer cells. The α-mangostin on MCF-10A cells showed no significant cytotoxicity until 5 μM	[83]
	10 μM/24 h	IF	α-Mangostin reduced the number of Ki-67 + cells in both MCF-7 and MDA-MB-231 cells	
	10 μM/24 h	BrdU	α-Mangostin reduced the incorporation of BrdU in MCF-7 cells	
MCF-7 (ER +) SK-BR-3 (HER-2 +) MDA-MB-231 (TN)	0,1.2, 2.5, 5.0, 10, 20 and 40 µM	MTT	α-Mangostin significantly inhibited cell proliferation in a concentration-dependent manner, with IC_50_ values of 9.69 µM for MCF-7 cells, 7.46 µM for SK-BR-3 cells, and 11.37 µM for MDA-MB-231 cells	[70]
SK-BR-3 (HER-2 +)	CME from pericarp of mangosteen (0–50 µg/mL for 48 h)		CME inhibited cell proliferation in a concentration-dependent manner, with an IC_50_ = 9.25 ± 0.64 µg/mL	[84]
BJMC3879 (murine mammary adenocarcinoma cells with a p53 mutation)	0, 4, 6, 8, 10, 12, 14, 16 and 18 µM	Cell Titer-Blue Cell Viability	α-Mangostin inhibited concentration-dependently the cell viability of BJMC3879 cells, with statistical significance from 8 µM	[85]
BJMC3879 luc2 (murine mammary adenocarcinoma cells transfected with luc2) MDA-MB-231 (TN)	0, 4, 8, 12, 16 and 20 µM/24 and 48 h	Cell Titer-Blue Cell Viability	The α-Mangostin inhibited the viability of BJMC3879 luc2 and MDA-MB-231 with IC_50_ values of 12 µM and 20 µM at 24 h, respectively	[78]
MDA-MB-468 (TN) AU565 (HER-2 +) SK-BR-3 (HER-2 +) T-47D (ER +)	7.5, 15 and 30 µM/24 h	MTT	α-Mangostin inhibited the cell proliferation of T-47D cells with an IC_50_ = 7.5 ± 0.5 µM; whereas those of SK-BR-3, MDA-MB-468 and AU565 were 23.88, 22.23 and 43.63 µM, respectively	[74]
	15 and 30 µM/3 and 6 h. After 14 days colonies were fixed, stained and counted	Crystal violet (2% w/v)	α-Mangostin inhibits T-47D colony formation in a concentration-dependent manner at 6 h of treatment with 15 and 30 µM	
HCC-1937 (TN)	0–100 µM/48 h	MTT	α-Mangostin inhibited cell proliferation, achieving IC_50_ values of 13 µM	[22]
MDA-MB-231 (TN)	0–48 µM 24 and 48 h	Cell-Titer-Blue Cell Viability	The α-Mangostin inhibited the cell viability of MDA-MB-231 with IC_50_ values of 20 µM and 16 µM at 24 and 48 h, respectively. Additionally, a decreased mRNA and protein expression of PCNA was observed with α-mangostin 20 µM	[77]
MDA-MB-231 (TN)	0, 6.25, 12.5, 25, 50, and 100 µM of α-mangostin or 0, 6.25, 12.5, 25, 50, and 100 µg/mL for 24 h	MTT	The α-mangostin and EEM pericarp inhibited cell viability in a concentration-dependent manner, with IC_50_ values of 23 µM and 26 µg/mL, respectively	[86]
MDA-MB-231 (TN)	0, 1, 3, 10, and 30 µM/24 and 48 h	XTT	α-Mangostin decreased cell proliferation and induced apoptosis in a concentration-dependent manner, with IC_50_ values of 4.8 ± 0.2 μM at 24 h and 2.2 ± 0.04 μM at 48 h. It was also less toxic to normal mammary epithelial cells	[87]
MDA-MB-231 (TN)	0–100 µM/24 h	CCK-8	α-Mangostin suppressed cell proliferation with an IC_50_ of 20 ± 1.3 μM. Cell viability decreased with α-mangostin concentrations higher than 25 μM	[88]
4T1 (TN)	1, 5, 10, 20, 40, 60, and 80 μM/24 h	MTT	The α-mangostin significantly reduced cell viability in a concentration-dependent manner, from 5 to 80 μM, with an IC_50_ = 13.98 µM. α-Mangostin IC_70_ = 18 µM induces mitochondrial ROS production in neoplastic cells	[89]
SUM-229PE (TN) MBCDF-D5 (TN) HCC1806 (TN) MBCDF (HER-2) T-47D (ER +)	0–10 µM for 6 days	SRB	The α-mangostin significantly inhibited the breast cancer cell proliferation in a concentration-dependent manner, with an IC_50_ of 3.13 ± 0.09, 0.77 ± 0.2, 2.59 ± 0.17, 5.23 ± 0.19 and 4.36 ± 0.17 μM for SUM-229PE, MBCDF-D5, HCC1806, MBCDF and T-47D respectively	[3]

*ER + * Estrogen Receptor- positive, *HER-2* Human Epidermal Growth Factor Receptor type 2 enriched, *TN* Triple Negative, *MRE* mangosteen rind extract, *MEM* methanolic extract of mangosteen, *CME* crude methanolic extract, *EEM* ethanol extract of mangosteen, *MTT* 3-(4, 5-dimethyltiazol-2-yl)-2,5-diphenyltetrazolium bromide, *XTT* 2,3-Bis(2-methoxy-4-nitro-5-sulfophenyl)-2H-tetrazolium-5-carboxanilide, *SRB* sulforhodamine B, inhibitory concentration at 50% (IC_50_), *CCK-8* Cell Counting Kit-8, *CR* cisplatin-resistant, *CCK-8* Cell Counting Kit, *IF* Immunofluorescence, *BrdU* bromodeoxyuridine, *PCNA* Proloferating Nuclear Antigen

**Fig. 3:**
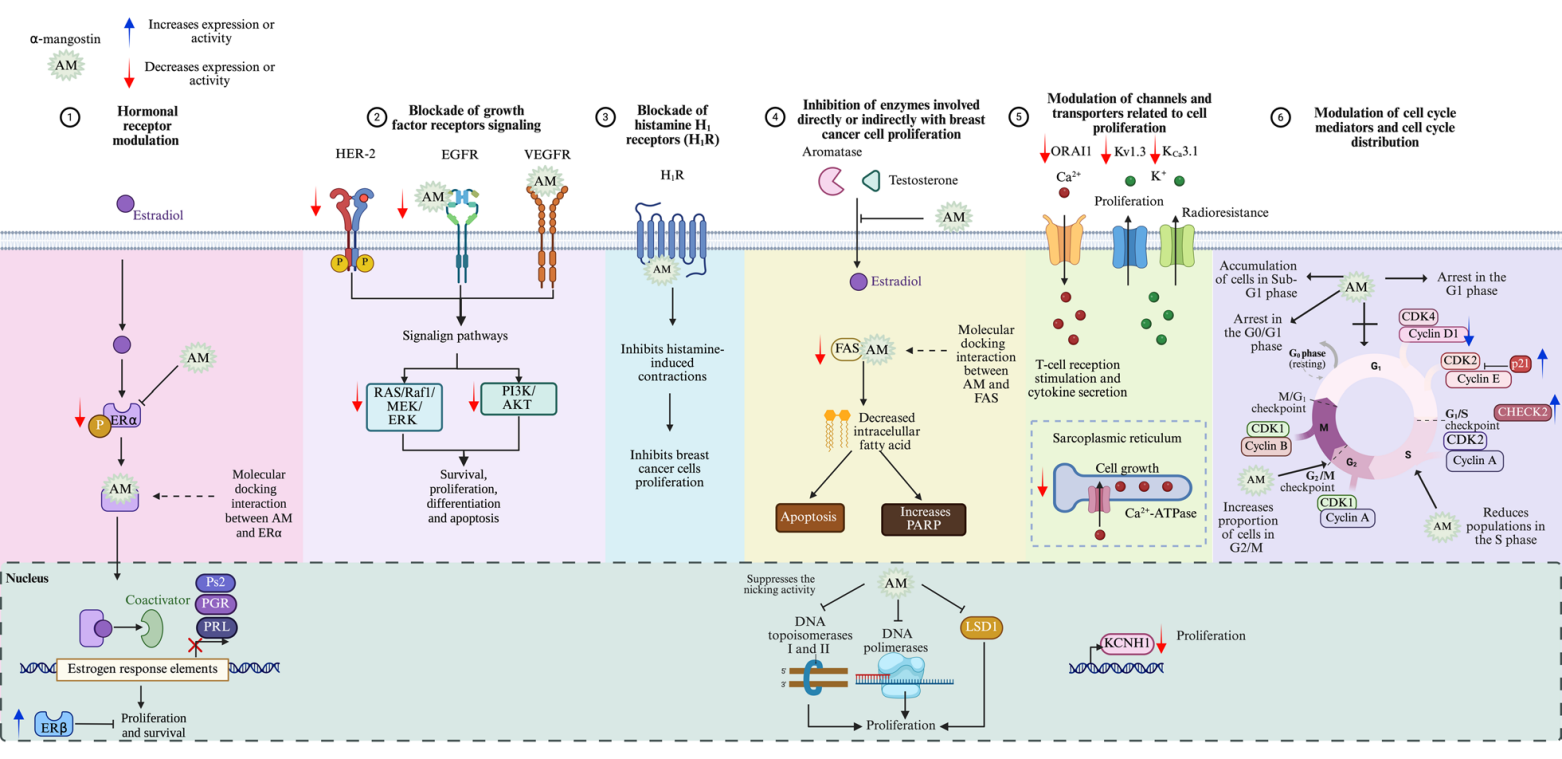
Antiproliferative mechanisms of α-mangostin. α-Mangostin (AM) exhibits diverse mechanisms to inhibit breast cancer cell proliferation, including: (1) Hormonal receptor modulation: AM reduces ERα expression and phosphorylation, increases ERβ levels, and inhibits estrogen-dependent gene expression. Docking studies indicate strong interactions with ERs, and derivatives of AM act as potential ER antagonists. (2) Blockade of growth factor receptor signaling: AM inhibits HER-2 phosphorylation and EGFR, and VEGFR binding, downregulating key oncogenic pathways (RAS/RAF1/MEK/ERK and PI3K/AKT). (3) Histamine H_1_ receptor (H_1_R) inhibition: AM blocks H_1_R, linked to tumor growth, and reduces breast cancer histamine levels. (4) Enzyme inhibition: AM targets aromatase, topoisomerases, DNA polymerases, Lysine-Specific Demethylase 1 (LSD1), and fatty acid synthase (FAS), disrupting proliferation, DNA processes, and lipid synthesis. (5) Ion channel modulation: AM inhibits oncogenic potassium channels (*KCNH1*, Kv1.3, KCa3.1), calcium transport (ORAI1, Ca^2+^ ATPase), impacting cell signaling and viability. (6) Cell cycle modulation: AM induces G1-phase arrest and downregulates cyclin-CDK complexes, preventing uncontrolled cell division. Figure created using BioRender.com

On the other hand, α-mangostin has demonstrated the ability to suppress breast cancer cell growth and the development of pre-neoplastic lesions induced by 7,12-dimethylbenzo (α) anthracene in a mouse mammary organ culture assay, with an IC_50_ of 2.44 µM [23]. Numerous studies highlight the impact of α-mangostin on breast cancer, identifying several mechanisms through which it may exert its antiproliferative effects (Fig. 3).

#### Modulation of hormone receptors by α-mangostin in breast cancer

In the canonical mechanism of action, ERα functions as a transcription factor upon ligand binding, driving the transcription of genes involved in cell proliferation. One mechanism through which α-mangostin inhibits cancer cell proliferation is by inhibiting ERα (Fig. 3). Specifically, at a concentration of 10 µM, α-mangostin suppresses ERα protein expression and decreases the estrogen-dependent gene Ps2 in MCF-7 cells. Notably, siRNA-mediated knockdown of ERα levels, followed by subsequent treatment with α-mangostin, reduces its effects, suggesting that the xanthone exerts its activity, at least in part, through ERα [76]. Additionally, ERα phosphorylation is reduced in T-47D cells treated with 30 µM of α-mangostin, suggesting that α-mangostin may additionally inhibit cell proliferation and survival by suppressing ERα signaling pathway [74]. Moreover, treatment of MCF-7 cells with mangosteen rind extract (50 and 100 µg/mL) decreased ERα gene expression in a concentration-dependent manner while increasing ERβ levels [80]. ERβ, in particular, plays a significant role in breast cancer, often acting as a tumor suppressor; while ERα promotes cancer cell proliferation, ERβ tends to inhibit it, and high levels of this protein are associated with better prognosis and improved survival rates [90].

Given the significance of estrogen signaling in breast cancer progression, blocking this pathway is a key therapeutic strategy in ER-positive tumors. In this context, it has been demonstrated that α-mangostin at 3.5 µM reduces the expression of estrogen-regulated genes, such as those encoding prolactin and PR, in MCF-7 cells, suggesting the interference with estrogen signaling [4].

In an additional study, the anti-proliferative effects of four xanthones, including α-mangostin, β-mangostin, mangostenol, mangaxanthone B, along with three benzophenones and one sterol, were evaluated. Initially, the antiproliferative activity of these compounds was assessed in the breast cancer cell lines MCF-7 and MDA-MB-231, identifying α-mangostin as the most potent compound, with IC_50_ values of 4.43 µM and 3.59 µM, respectively. Subsequently, a molecular docking simulation study was conducted to evaluate the interactions of xanthones with 2OCF (Protein Data Bank ID for the protein structure of ER). The analysis regarding α-mangostin revealed key interactions contributing to its potential biological activity. Specifically, the prenyl group at C8 of α-mangostin interacts with the Leu320, Trp393, Phe445, and Val446 residues of 2OCF at various distances (Å) through hydrophobic interactions. The aromatic ring A of α-mangostin interacts with Arg394, while rings B and C with Ile326 and Arg394 through hydrophobic interactions. Furthermore, the hydroxyl group at C1 and the carbonyl group (C = O) interact with Trp393 through hydrogen bonding. Additionally, a molecular docking simulation of xanthones was performed with 4PIV (Protein Data Bank ID for the β-ketoacyl part of Fatty Acid Synthase, FAS), which will be discussed in detail in the section regarding FAS inhibition [19].

Other studies have also explored α-mangostin and its derivatives as potential ERα antagonists. In one of them, α-mangostin was chemically modified by substituting the hydroxyl group at C6 with benzoyl derivatives. Computational analyses predicted that the resulting derivatives AMB-1, AMB-2, and AMB-10 may function as potential ERα antagonists. Of note, these findings were based solely in silico approaches and were not validated through experimental assays [71]. More recently, another study explored the conjugation of glycine to the hydroxyl groups at positions C3 and/or C6 of the α-mangostin, leading to the development of glycine-conjugated α-mangostin derivatives. Among these, the Am1Gly, with glycine attached to the hydroxyl group at C3, demonstrated promising potential as an ERα antagonist. Further pharmacophore modeling revealed that Am1Gly1 and Am2Gly2 possess structural features comparable to those of active drugs targeting ERα, reinforcing their potential for therapeutic development. Molecular docking analyses indicated that Am1Gly1 interacts with key residues essential for functioning as a 4-hydroxytamoxifen-like ERα antagonist, exhibiting dynamic interactions similar to those observed with 4-hydroxytamoxifen. These findings were supported by molecular dynamics simulations, which confirmed consistent amino acid variation patters over 200 ns. Additionally, binding affinity analysis, molecular docking, dynamic simulations, and pharmacophore modeling collectively supported the potential of Am1Gly1 as an ERα antagonist. This study was not accompanied by experimental validation [91].

#### Inhibition of growth-factor receptors signaling by α-mangostin in breast cancer

HER-2 is a receptor with intrinsic tyrosine kinase activity, comprising an extracellular ligand-binding domain, a transmembrane domain, and an intracellular tyrosine kinase domain. While HER-2 does not have a known ligand that directly binds to it, it acts as the preferred heterodimerization partner for other members of the epidermal growth factor receptor (EGFR) family (ErbB). Thus, upon ligand binding to other ErbB members, HER-2 is recruited, leading to the autophosphorylation of its intracellular tyrosine kinase domains. This activation triggers the phosphatidylinositol 3-kinase (PI3K)/protein kinase B (PKB, also known as AKT) and mitogen-activated protein kinase (MAPK) signaling pathways, which are crucial for processes closely related to breast tumorigenesis such as cell survival, proliferation, differentiation, and apoptosis [92]. Notably, treatment of T-47D breast cancer cells with 30 µM of α-mangostin resulted in reduced phosphorylation of HER-2 at the Tyr1221/1222 within 30 min, leading to the deactivation of RAS/RAF1/MEK/ERK and PI3K/AKT signaling pathways, and consequently, an anti-proliferative and pro-apoptotic effect (Fig. 3) [74].

Additionally, molecular docking analysis provided evidence that garcinone E, another prenylated xanthone, which differs from α-mangostin by an isoprenyl group at the C5 position and hydroxyl group at the C7 position (Fig. 2), binds tightly to the EGFR and the vascular endothelial growth factor receptor (VEGFR), inhibiting their kinase activity with IC_50_ values of 315.4 nM and 158.2 nM, respectively. Furthermore, garcinone E blocks the endothelial growth factor (EGF)- and vascular endothelial growth factor (VEGF)-induced phosphorylation of EGFR and VEGFR, respectively. Although α-mangostin also binds to these receptors, it does so with less affinity than garcinone E. Beyond its molecular interactions, garcinone E demonstrated potent anti-cancer activity, inhibiting cancer cell proliferation, endothelial cell migration, invasion, and tube formation. Its dose-dependent suppression of angiogenesis was confirmed in ex vivo and in vivo models. In an MDA-MB-231 xenograft model, garcinone E (2 mg/kg) significantly reduced tumor growth, weight, and microvessel density, correlating with downregulation of VEGFR2, EGFR, and Ki-67 [93]. However, mangosteen rind extract (50 and 100 µg/mL) decreased the gene expression of EGFR in MCF-7 cells in a concentration-dependent manner [80]. In the TN breast cancer phenotype, EGFR is highly expressed, which is associated with cancer progression, proliferation, metastasis, and drug resistance, making EGFR a promising pharmacological target [94]. Therefore, α-mangostin could be considered a potential compound for targeting this receptor.

#### Inhibition of H_1_R by α-mangostin in breast cancer

α-Mangostin has been identified as an inhibitor of the H_1_R. Specifically, it exhibits a concentration-dependent inhibition of [^3^H]mepyramine binding, a selective H_1_ receptor antagonist, in rat aortic smooth muscle cells. Kinetic analysis indicates that α-mangostin acts as a competitive inhibitor of the H_1_R, preventing [^3^H]mepyramine from binding effectively with an IC_50_ value of 2.27 µM. Scatchard plot analysis further revealed that [^3^H]mepyramine binds to a high affinity receptor site with a dissociation constant (K_d_) of 11.72 nM and a maximum binding capacity (B_max_) of 275.95 fmol/mg. Treatment with α-mangostin increased the K_d_ value to 38.02 nM, indicating reduced affinity, while the Bmax value remained unchanged. These findings confirm that α-mangostin function as a specific H_1_R antagonist [33] (Fig. 3). This is relevant because there is a direct correlation between endogenous histamine levels and breast cancer development. In particular, higher histamine concentrations have been observed in tumors and adjacent tissue of breast cancer patients compared to the unaffected tissue of healthy individuals [95]. Additionally, there is a significant increase in histamine levels in the plasma and tissues of patients with ductal breast cancer. The plasma histamine concentrations in women with ductal breast carcinoma depend on the number of involved lymph nodes and the grade of histologic malignancy [96]. Additionally, H_1_R is overexpressed in basal-type and HER-2-enriched breast tumors, which correlates with a shorter overall survival [97]. This supports the idea that histamine and its receptors are involved in cell growth. Furthermore, several antihistamines have been shown to inhibit breast cancer cell proliferation both in vitro and in vivo [98–101].

#### Inhibition of enzymatic activities associated with breast cancer cell proliferation by α-mangostin

##### Aromatase.

Aromatase plays a pivotal role in converting androgens into estrogen through aromatization. Notably, breast cancer tissues exhibit higher levels of aromatase compared to healthy tissues [102]. Consequently, one of the therapeutic strategies in the clinical practice involves aromatase inhibitors to reduce breast cancer growth. These inhibitors hamper the enzyme’s function, affecting estrogen synthesis and, consequently, blocking breast cancer cell proliferation. Interestingly, a non-cellular, enzyme-based microsomal assay, revealed that garcinone D, garcinone E, α-mangostin, and γ-mangostin exhibited a dose-dependent aromatase inhibitory activity. Among these compounds, garcinone D (IC_50_ = 5.2 µM) and γ-mangostin (IC_50_ = 6.9 µM) demonstrated the strongest inhibitory effects in this assay. In contrast, the two other xanthones, α-mangostin (IC_50_ = 20.7 µM) and garcinone E (IC_50_ = 25.1 µM), displayed moderate inhibition of aromatase activity [103] (Fig. 3).

##### DNA polymerases and topoisomerases.

DNA polymerase synthesizes new DNA strands during replication, while topoisomerases I and II play critical roles in cutting single-stranded or double-stranded DNA to relieve tension during replication or transcription events [104]. Interestingly, α-mangostin exhibits a strong inhibitory effect on both DNA polymerases (IC_50_ values 14.8 µM–25.6 µM) and topoisomerases I and II (15.0 µM and 7.5 µM, respectively). Notably, α-mangostin completely suppresses the nicking activity of topoisomerase I at concentrations greater than 20 µM, while at 10 µM, it fully inhibits topoisomerase II. Interestingly, as determined by thermal transition analysis, α-mangostin does not directly bind to double-stranded DNA. This suggests that the decreased proliferation observed with α-mangostin may be partially due to the inhibition of these enzymes [105] (Fig. 3).

##### Lysine-specific demethylase 1 (LSD1).

LSD1 is an enzyme that plays a crucial role in gene expression regulation through histone modification. It is frequently overexpressed in several human cancers, including breast cancer, where it promotes tumor progression by supporting cancer cell survival and creating a pro-oncogenic environment. As a histone demethylase, LSD1 removes methyl groups from specific lysine residues on histone proteins, mainly histone H3 at lysine 4 (H3K4) and lysine 9 (H3K9). This chromatin remodeling process, directly influences transcriptional activity, either repressing or activating gene expression [106]. In ERα-positive breast cancer, LSD1 demethylates H3K9 at ERα target genes in an estrogen-dependent manner, facilitating transcriptional activation. This regulatory mechanism is reinforced by Proline, Glutamate, and Leucine Rich Protein 1 (PELP1), a co-activator that links LSD1 to ERα, forming the LSD1-PELP1-ERα axis implicated in hormone resistance. Additionally, LSD1 indirectly promotes *CYP19A1* expression, encoding aromatase, through its interaction with HER-2 signaling [107]. Conversely, LSD1 demethylates non-histone proteins, such as p53, leading to the repression of its activity [108]. Moreover, LSD1 downregulates *CDH1* expression, the gene encoding E-cadherin, which may result in decreased E-cadherin levels, compromising cell adhesion, thereby facilitating EMT, a critical step in metastasis [109]. This dual regulatory function underscores LSD1´s central role in tumor progression and endocrine resistance, making it a promising therapeutic target. Regarding this, α-mangostin is a specific inhibitor of LSD1, with an IC_50_ value of 2.81 ± 0.44 µM. Bioactivity studies and molecular docking analysis indicated that this xanthone could inhibit the migration and invasion of MDA-MB-231 cells by targeting intracellular LSD1 activity. Moreover, α-mangostin increases the expression of the epithelial cell marker E-cadherin, while downregulating the mesenchymal cell marker N-cadherin, indicating its potential role in modulating EMT [110] (Fig. 3).

##### FAS inhibition.

FAS is a metabolic enzyme synthesizing long-chain saturated fatty acids, essential for membrane formation in proliferating cells. In cancer cells, including breast cancer, FAS overexpression leads to de novo lipogenesis, incorporating lipids into the lipid rafts of tyrosine kinase membrane receptors, such as the EGFR family, triggering oncogenic signaling pathways that promote cell survival, proliferation, migration, and invasion. Therefore, inhibiting FAS reduces phospholipid synthesis and cell proliferation while increasing apoptosis. Additionally, FAS inhibition can disrupt lipid raft assembly and impair EGFR localization to the membrane of breast cancer cells. Due to its extensive and high expression in many human cancers, FAS has been proposed as a potential molecular target for developing antineoplastic drugs. In this context, α-mangostin (1–4 µM) has been shown to suppress the expression and activity of FAS in MCF-7 and MDA-MB-231 cells, leading to a decreased intracellular fatty acid accumulation (Fig. 3). This reduction can lower cell viability, induce apoptosis in breast cancer cells, increase levels of the Poly (ADP-ribose) polymerase (PARP) activation product, and affect the balance between anti-apoptotic BCL-2 and pro-apoptotic BAX proteins [75]. Additionally, treating breast cancer cells with α-mangostin decreases the phosphorylation of AKT, which suggests that PI3K/AKT is related to the downregulation of FAS expression by the xanthone. A molecular docking simulation study of α-mangostin with 4PIV determined that different groups of α-mangostin interact with several residues of 4PIV. The aromatic ring A of α-mangostin interacts with Leu1971 and Ile2063 through hydrophobic interactions. The hydroxyl groups at C1, C3, and C6 form hydrogen bonds with Ile 2068, Leu1971, and Trp2060 residues of 4PIV. The prenyl group at C2 interacts with Val1973 and Ile2068 residues, while the prenyl group at C8 interacts with Leu1975, Tyr2034, and Leu2069 residues through hydrophobic interactions. The methoxyl group at C7 interacts with Ser2021 and Gly2061 residues through hydrogen interactions. Additionally, ring C and the C = O interact with Ile2068 through hydrophobic and hydrogen interactions, respectively. These findings were supported by cell proliferation experiments, which demonstrated that α-mangostin exhibited strong anti-proliferative activity against breast cancer cell lines MCF-7 and MDA-MB-231, with IC_50_ values of 4.43 µM and 3.59 µM, respectively [19].

#### Modulation of ion channels by α-mangostin in breast cancer

The oncogenic voltage-gated potassium channel subfamily H member 1 (*KCNH1*), encodes the Ether-à-go-go 1 (EAG1, Kv10.1) potassium channel, which is overexpressed in various cancers, while inhibiting its expression or activity decreases cancer cell proliferation [111]. Interestingly, α-mangostin at concentrations of 3.5 µM and 7 µM significantly reduced *KCNH1* gene expression in MCF-7 and T-47D breast cancer cells, respectively [4] (Fig. 3). Additionally, it was demonstrated that α-mangostin decreased *KCNH1* gene expression in cervical cancer cells, both in vitro and in vivo [43]. These findings suggest that inhibiting *KCNH1* expression could be another mechanism by which α-mangostin decreases cancer cell proliferation.

Furthermore, cytosolic calcium levels regulate cell growth. It has also been demonstrated that α-mangostin decreases the activity of Ca^2+^-ATPase and the calcium transport of the sarcoplasmic reticulum from rabbit skeletal muscle in a concentration-dependent manner, with an IC_50_ of 5 µM [112]. Additionally, the generation of intracellular calcium signaling is crucial for T-cell receptor stimulation and cytokine secretion. This signaling is mediated by the calcium release-activated calcium modulator 1 (ORAI1) channel and potassium ion channels, which provide the electrical driving force to generate sufficient calcium ion influx. α-Mangostin inhibited ORAI1 in a concentration-dependent manner with an IC_50_ of 1.27 ± 1.144 µM, and at 3 µM, suppressed the activity of the voltage-dependent potassium channel Kv1.3 and the intermediate-conductance calcium-activated K_Ca_3.1 by 41.38 ± 6.19 % and 51.16 ± 5.38 %, respectively [113]. Noteworthy, the overexpression of Kv1.3 and K_Ca_3.1 channels is also associated with aberrant cancer cell proliferation; indeed, the overexpression of Kca3.1 confers radioresistance to breast cancer cells [114, 115].

Although studies on α-mangostin modulation of ion channels in cancer are limited, an electrophysiological investigation explored its analgesic effects through ion channels modulation in dorsal root ganglion neurons. Specifically, α-Mangostin (1–3 µM) influenced neuronal excitability by hyperpolarizing the resting membrane potential, likely due to an increased K^+^ conductance, which ultimately suppressed action potential generation. This xanthone activated TREK-1, TREK-2, and TRAAK channels. Additionally, at 0.43 ± 0.27 µM, α-mangostin inhibited capsaicin-induced TRPV1 currents and partially reduced tetrodotoxin-sensitive voltage-gated Na^+^ (Na_v_) channel activity. Molecular docking analyses suggest that α-mangostin´s oxygen atoms establish hydrogen bonds with these channels [116].

#### Modulation of the cell cycle by α-mangostin in breast cancer

The inhibition of cell proliferation is closely related to cell cycle blockade. Numerous cell cycle mediators, including cyclins, CDKs, and CDK inhibitors (CDKI), play a crucial role in controlling different phases of the cell cycle. Disruption of their balance can alter cell cycle distribution, finally affecting cell proliferation [117].

In this context, α-mangostin (20 µM) led to G1-phase cell cycle arrest in MDA-MB-231 cells by increasing the expression of p21, a CDK inhibitor. Additionally, there was a tendency for increased expression of the cell cycle checkpoint regulator CHEK2. This rise in p21 and CHEK2 expression decreased CDKs and cyclin expression, resulting in G1-phase arrest and inhibition of cell proliferation [77]. In another study involving MDA-MB-231 cells, treatment with 5 µM α-mangostin resulted in an increased proportion of cells in the G2/M and S phases, while the proportion of cells in the G1-phase decreased. This effect was also associated with decreased Cyclin D1 expression (*CCND1*) [70]. Similarly, treatment with of α-mangostin (3.5 µM and 7.1 µM) in MCF-7 and T-47D cells reduced *CCND1* gene expression [4].

The active complex CDK4/Cyclin D1 phosphorylates retinoblastoma (Rb) protein, which releases the transcription factor E2F and activates the expression of GI/S phase genes. Therefore, the up-regulation of the CDK4/Cyclin D1-Rb pathway increases the mitogenic potential of neoplastic cells, contributing to resistance to endocrine therapy in hormone-dependent cancers, including breast cancer. Interestingly, it has been determined, by a cell-free biochemical assay, that α-mangostin (IC_50_ value of 8.5 µM), as well as other isoprenylated xanthones, inhibited CDK4/Cyclin D1 complex, preventing the phosphorylation of downstream targets, therefore inhibiting uncontrolled cell cycle progression [22].

α-Mangostin at 1 µM, 5 µM and 10 µM resulted in a concentration-dependent accumulation of cells in the Sub-G1 phase, indicating xanthone-induced apoptosis in MCF-7 cells [76]. In T-47D cells, similar effects were observed, treatment with α-mangostin at 15 µM and 30 µM promoted Sub-G1 phase arrest with a decrease in G1-phase cells [74]. Additionally, α-mangostin treatment at 4.36 µM and 5.23 µM significantly promoted sub-G1 accumulation of T-47D and MBCDF cells, respectively, indicating cell death [3].

In BJMC3879 and BJMC3879 luc2 breast cancer cells, the treatment with α-mangostin 8 µM and 12 µM, respectively, induced cell cycle arrest in the G1 phase, with reduced populations in the S phase [78, 85].

### α-Mangostin-induced apoptosis in breast cancer cells: mechanism of action

Apoptosis is a tightly regulated process of programmed cell death characterized by morphological changes in cells, chromatin condensation, DNA fragmentation, and caspase activation. It occurs via two primary pathways: the extrinsic and the intrinsic pathways. The extrinsic pathway is initiated by ligand binding, including Tumor Necrosis Factor-alpha (TNF-α), CD95L/FasL, or TRAIL to their corresponding death receptors on the cell surface. This interaction leads to the formation of death-inducing signaling complexes and activation of caspase-8, which initiates the apoptotic cascade. In contrast, the intrinsic pathway, or the mitochondrial pathway, is triggered by cellular stress or DNA damage. This pathway involves mitochondrial membrane depolarization and is regulated by members of the BCL-2 protein family. Disruption of the mitochondrial membrane leads to the release of key apoptotic factors into the cytosol, including the cytochrome c, a caspase-dependent apoptotic factor, and Apoptosis-Inducing Factor (AIF), a caspase-independent apoptotic factor. Both pathways converge on the activation of caspase-3, cleavage of PARP, DNA fragmentation, and, ultimately, apoptotic cell death. Apoptosis is regulated by a variety of signaling pathways, PI3K/AKT, MAPK/ERK1/2, JNK1/2, and p38 play pivotal roles in regulating cellular fate. ERK1/2 promotes cell growth, survival, and differentiation, whereas JNK1/2 and p38 are primarily associated with apoptotic processes. The PI3K/AKT pathway is another significant regulator of apoptosis, with AKT influencing key proteins such as BCL-2, BAX, and caspase-3 [74].

It has been demonstrated that one of the primary mechanisms by which α-mangostin exerts its effects in cancer cells is the induction of apoptosis through distinct pathways. The pro-apoptotic effects of α-mangostin has been predominantly investigated in the human breast cancer cell lines MCF-7, T-47D, and MDA-MB-231 cells, as summarized in Table 3.

**Table 3:** Apoptotic effects of α-mangostin or mangosteen extract on human breast cancer cell lines

Breast cancer cell line	α-Mangostin concentration	Results	Ref
MCF-7	1, 5, and 10 µM	α-Mangostin induced chromatin condensation and apoptotic body formation, along with a concentration-dependent accumulation of cells in Sub-G1 phase. At 10 µM, this apoptotic response was marked by the activation of caspasas-8, -9, and -7, and cleavage of PARP. This process was associated with an increase in BAX and p53 protein levels, while BID and BCL-2 protein expression were reduced. Additionally, cytochrome c and AIF were released from mitochondria into the cytosol	[76]
	10, 20, and 30 µM	α-Mangostin induced concentration-dependent apoptotic cell death by activating MOAP-1, a tumor suppressor that interacts with activated BAX, promoting its oligomerization and downregulation of BCL-X_L_. As a result, mitochondrial dysfunction occurs, followed by cytochrome c release, caspase activation, and ultimately, apoptosis	[82]
MCF-7 MDA-MB-231	1, 2, and 4 µM	α-Mangostin induced a pronounced, concentration-dependent increase in PARP cleavage, accompanied by downregulation of the anti-apoptotic protein BCL-2 and upregulation of pro-apoptotic BAX. Additionally, a notable decrease in phosphorylated AKT levels was observed. This findings suggest that the PI3K/AKT signaling pathway may be involved in the α-mangostin-mediated down-regulation of FAS expression. Apoptosis exhibited a concentration-dependent pattern, with marked increase at 4 µM	[75]
MCF-7 SK-BR-3 MDA-MB-231	5, 10, and 15 µM	α-Mangostin induced PARP cleavage in a concentration-dependent manner following 20 h of treatment. Additionally, treatment with 10 µM α-mangostin led to a time-dependent increase in PARP cleavage in MCF-7 cells	[70]
T-47D	15 and 30 µM	Treatment with α-mangostin at 15 and 30 µM, over different time points resulted in pronounced DNA fragmentation and a concentration-dependent increase in the sub-G1 cell population, rising from 3.3% in control conditions until 21% upon the highest treatment. At 30 µM, cells exhibited hallmark apoptotic features, including vacuolization, shrinkage, rounding, and chromatin condensation, accompanied by mitochondrial dysfunction. This apoptotic response was associated with and elevated BAX/BCL-2 ratio and downregulation of the anti-apoptotic protein MCL-1. Additionally, α-mangostin stimulated the expression of pro-apoptotic transcription factor CHOP and c-Jun, highlighting its capacity to modulate multiple apoptotic signaling pathways. Molecular events included cytochrome c release into the cytosol and activation of caspases -3 and -9. Concurrently, α-mangostin reduced the phosphorylation of HER-2, c-Raf, ERK 1/2, and PI3K/AKT, while enhancing JNK1/2 and p38 phosphorylation	[74]
MDA-MB-231	20 µM	α-Mangostin induced mitochondrial apoptosis by increasing the activity of caspases-3, -8, and -9, while reducing mitochondrial cytochrome c levels	[77]
	2, 5, 10, and 15 µM	α-Mangostin activated caspasa-3 in a concentration-dependent manner within the 2–15 µM range. It also upregulated BAX and downregulated BAD expression at concentration of 5–10 µM. Notably, phosphorylated AKT levels declined from 10 µM onward. These pro-apoptotic effects are mediated through inhibition of AKT signaling	[70]

*BAX* BCL-2-associated X protein, *BID* BH3-interacting domain death agonist, *BCL-2* B-cell lymphoma 2, *AIF* Apoptosis Induction Factor, *MOAP-1* Modulator of Apoptosis, *BCL-X*_*L*_ B-cell lymphoma-extra large, *AKT* Protein Kinase B, *PI3K* Phosphatidylinositol 3-kinase, *FAS* Fatty acid synthase, *PARP* Poly (ADP-ribose) polymerase, *MCL-1* myeloid Cell Leukemia 1, *CHOP* C/EBP Homologous Protein, *HER-2* Human Epidermal Growth Factor Receptor type 2 enriched, *c-Raf* RAF proto-oncogene serine/threonine-protein kinase, *ERK 1/2* Extracellular Signal-Regulated Kinase 1 and 2

In MDA-MB-231 cells, prenylated xanthones from mangosteen pericarp, including α-mangostin, γ-mangostin, and garcinone E, target mitochondria by inhibiting mitochondrial respiratory chain complex II. This inhibition disrupts oxidative mitochondrial respiration, increases mitochondrial proton leakage, and diminishes ATP production. As a result, mitochondrial superoxide levels rise, leading to membrane permeabilization and trigger apoptosis through caspase-3 and -7 activation. Therefore, the apoptotic effects of this xanthones are mediated through interference with mitochondrial respiration and cellular energy metabolism [87].

Likewise, in SK-BR-3 cells, treatment with crude methanolic extract (CME) from mangosteen pericarp (20–100 µg/mL) resulted in distinct morphological alterations, such as cell retraction, rounding, and membrane blebbing, accompanied by the formation of apoptotic bodies, nuclear fragmentation, and shrinkage. Notably, at 100 µg/mL, CME induced significant DNA fragmentation, further indicating its pro-apoptotic activity [84].

Moreover, α-mangostin induces apoptosis in additional cancer cell models through distinct mechanisms. In BJMC3879 murine mammary adenocarcinoma cells, α-mangostin increased the number of TUNEL-positive cells, elevated caspase activity, and reduced mitochondrial membrane potential [85]. At a concentration of 12 µM for 24 h, α-mangostin triggered apoptosis in BJMC3879 luc2 cells by activating caspases -3, -8, and -9. At 48 h, a significant increase in apoptotic cells was observed via TUNEL assays, and α-mangostin also enhanced the release of cytochrome c into the cytosol [78].

**Fig. 4:**
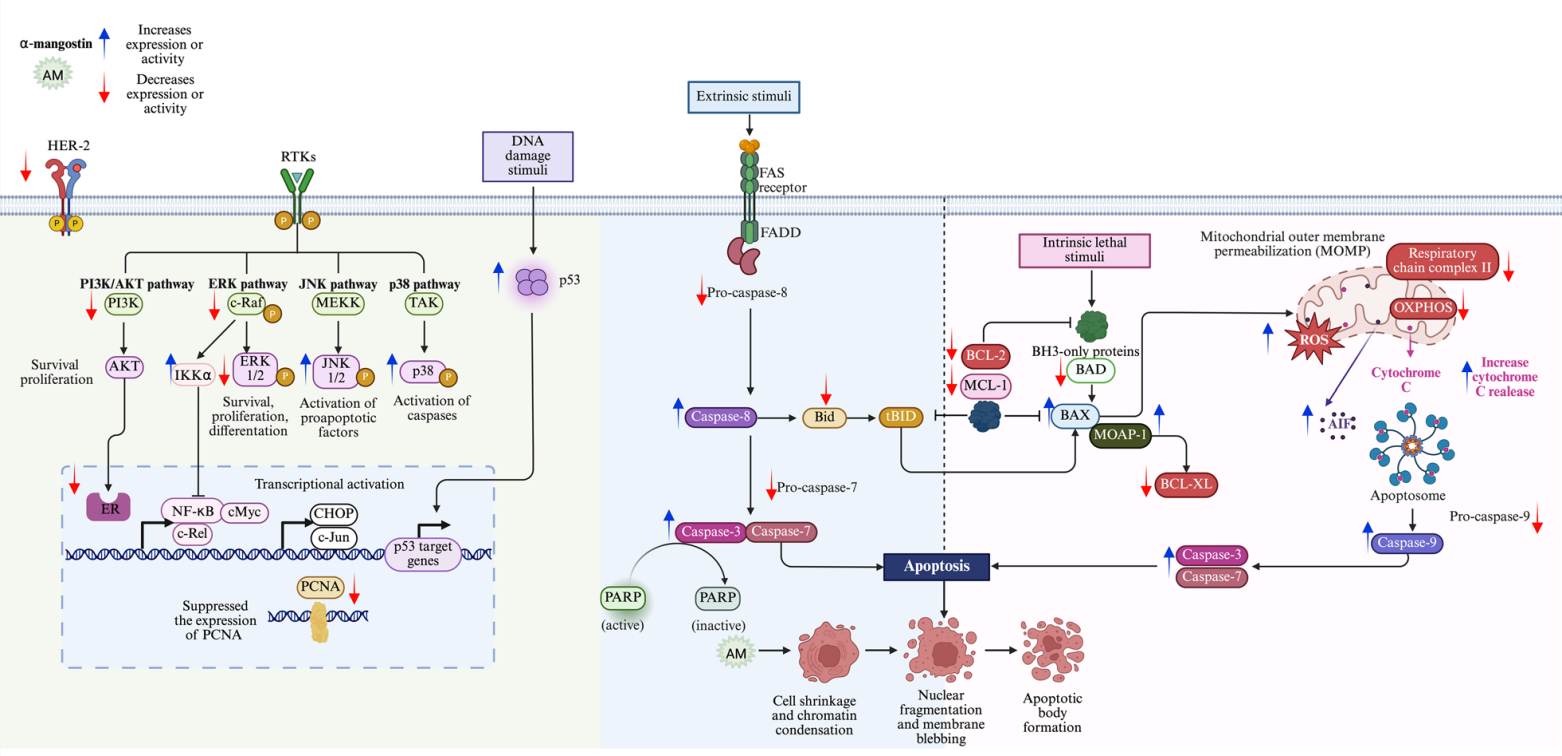
Antiapoptotic mechanisms of α-mangostin. The xanthone α-mangostin (AM) triggers apoptosis via the extrinsic pathway, initiated by ligand-receptor interactions leading to caspase-8 activation, and the intrinsic pathway, which involves mitochondrial membrane depolarization, cytochrome c release, and caspase-9 activation. Both pathways converge on the activation of caspase-3, cleavage of PARP, and execution of apoptosis. AM regulates BCL-2 family protein, downregulating BCL-2, BID, MCL-1, BCL-XL, and BAD, and upregulating BAX. Also, AM affects apoptosis through an independent caspase pathway, including apoptosis-inducing factor (AIF). Additionally, AM downregulates AKT and ERK1/2 signaling while enhancing JNK1/2 and p38 phosphorylation, favoring apoptotic cell death. AM-induced apoptosis is mediated by the Modulator of Apoptosis 1 (MOAP-1), which interacts with activated BAX while downregulating BCL-XL. AM also increases p53 expression, targets mitochondrial respiratory chain complex II, inhibits oxidative mitochondrial respiration (OXPHOS), and increases ROS production. Figure created using BioRender.com

In a general way, α-mangostin induces apoptosis through both caspase-dependent and caspase-independent mitochondrial pathways, and is also associated with the modulation of signaling pathways involved in cell death regulation (Fig. 4).

### Mechanisms of α-mangostin-mediated inhibition of cell adhesion, invasion and metastasis in breast cancer

The inhibitory mechanism of α-mangostin in these processes include the suppression of EMT, a biological process in which epithelial cells acquire mesenchymal characteristics, leading to reduced adhesion and enhanced migratory and invasive potential. While EMT plays a crucial role in embryonic development and wound healing, its dysregulation is closely linked to cancer metastasis. Another key mechanism involves the inhibition of FAK, a protein that regulates cell proliferation, survival, adhesion, and migration. The overexpression of FAK in cancer is associated with abnormal cell invasion and metastasis.

#### Inhibition EMT in breast cancer by α-mangostin

EMT is marked by decreased expression of E-cadherin and increased expression of N-cadherin and vimentin, along with cellular proteases. The primary mediators of the EMT include signaling through Tumor Growth Factor β (TGF-β), Notch, and Wnt, and is also influenced by the tumor microenvironment, such as hypoxia and differential expression of micro RNAs (miRNAs). These signaling mechanisms converge on transcription factors like Snail, Zeb, and Twist, whose differential expression in tumors has been shown to lead to EMT [118].

Interestingly, in MCF-7 cells, the mangosteen rind extract (50 and 100 µg/mL) increased the gene expression of E-cadherin while decreasing the levels of N-cadherin and Snail in a concentration-dependent manner, as well as inhibiting the activity of metalloproteinase-9 (MMP-9) and reducing the migration of these cells [80], strongly supporting its EMT-inhibitory capacity (Table 4).

**Table 4:** Mechanisms of α-mangostin inhibiting metastasis and angiogenesis in breast cancer and endothelial cells

Molecular target or mechanism	Effect of α-mangostin	Study type	Model or cell line used	Ref
EMT markers (E-cadherin, N-cadherin, Snail)	Mangosteen ↑ E-cadherin, ↓ N-cadherin and Snail expression, reducing migration	In vitro	MCF-7	[80]
MMP-2, MMP-3, MMP-9	↓ activity and expression, reduces adhesion, motility, invasion, migration	In vitro	MCF-7	[79, 80]
ERK/NF-κB/AP-1 (c-Fos, c-Jun)	↓ phosphorylation ERK and DNA binding activity of NF-kB, AP-1, c-Fos, and c-Jun, inhibits MMP expression	In vitro	MCF-7	[79, 119]
IκK/IκBα	↑ IκK expression, blocks IκBα phosphorylation, further inhibits MMPs	In vitro	MCF-7	[79]
STAT3	Binds and inhibits STAT3, reducing migration and invasion	In vitro, in silico	MCF-7 MDA-MB-231	[83]
CXCR4	↓ migration through interaction with CXCR4 (molecular docking)	In vitro, in silico	MDA-MB-231	[88]
FAK	Inhibits Tyr397 phosphorylation, reduces interaction with ECM proteins; effect linked to FAS inhibition	In vitro	MCF-7, MDA-MB-231	[75]
VEGFR-2 (Y1175 phosphorylation)	Inhibits phosphorylation, suppresses HUVEC and HUAEC proliferation, migration, and tubule formation	In vitro	HUVEC, HUAEC	[121]
ROS production	Reduces ROS under hypoxic conditions, blocks VEGF-induced endothelial responses (proliferation, migration, and tube formation)	In vitro	REC (retinal endothelial cells)	[122]
MAPK/ERK1/2 pathway	Suppresses VEGF-induced MAPK/ERK1/2 activation	In vitro	REC	[122]
VEGF protein expression	Mangosteen reduces VEGF expression	In vitro	T-47D	[123]
Tumor microvessel density	Panaxanthone and α-mangostin reduce microvessel density in tumors	In vivo	BJMC3879, BJMC3879luc2 (murine xenografts)	[78, 85]
AKT phosphorylation (Thr308)	Inhibits phospho-AKT, suggests downstream suppression of angiogenesis-related signaling	In vitro, In vivo	BJMC3879, BJMC3879luc2	[78, 85]

*FAK* Focal Adhesion Kinase, *FAS* Fatty Acid Synthase, *EMT* Epithelial-Mesenchymal Transition, *MMP* Matrix Metalloproteinase; *CXCR4* C-X-C Chemokine Receptor Type 4, *STAT3* Signal Transducer and Activator of Transcription 3, *VEGF* Vascular Endothelial Growth Factor, *VEGFR-2* Vascular Endothelial Growth Factor Receptor 2, *HUVEC* Human Umbilical Vein Endothelial Cells; *HUAEC* Human Umbilical Artery Endothelial Cells, *REC* Retinal Endothelial Cells, *PLCγ* Phospholipase C gamma, *MAPK* Mitogen-Activated Protein Kinase, *ERK* Extracellular Signal-Regulated Kinase; *AKT* Protein Kinase B, *eNOS* Endothelial Nitric Oxide Synthase, *IκBα* Inhibitor of NF-kappa-B alpha, *IκK* IκB kinase, *NF-κB* Nuclear Factor Kappa-light-chain-enhancer of activated B cells, *AP-1* Activator Protein 1

Excessive degradation of the extracellular matrix is a hallmark of tumor invasion and migration, often involving overexpression of MMP-2 and MMP-9. In MCF-7 breast cancer cells, at 6 µM, α-mangostin inhibited cell adhesion by 61% and cell motility by 90%. Furthermore, α-mangostin reduced cell invasion and migration by 55% and 61%, respectively, by changing cell shape and reducing their invasive and migratory capacity. α-Mangostin antimetastatic effects are due to the inhibition of MMP-2 and MMP-9 activity, reducing their expression at both mRNA and protein levels, achieving inhibition rates of 88% for MMP-9 and 95% for MMP-3 at 6 µM. Mechanistically, α-mangostin inhibits ERK phosphorylation and reduces the DNA binding activity of NF-kB, AP-1, c-Fos, and c-Jun, key transcription factors involved in TPA-induced MMP expression. α-Mangostin also increases the expression of the kinase inhibitor IkK, blocking phosphorylation of IkBα, which further suppresses MMP expression. Combining α-mangostin with IkK inhibitors demonstrates a synergistic reduction in MMP levels [79]. Additionally, α-mangostin inhibits the TNF-α induced NF-kB translocation into the nucleus [119] (Table 4).

Moreover, the Signal Transducer and Activator of Transcription 3 (STAT3) is a transcription factor that, when activated, promotes EMT by inducing the expression of EMT-related genes. STAT3 is also involved in various signaling pathways that contribute to breast cancer progression, promoting proliferation, inhibiting apoptosis, supporting angiogenesis and metastasis, and conferring resistance to breast cancer therapy [120]. Interestingly, α-mangostin binds to STAT3, inhibiting MCF-7 and MDA-MB-231 cells migration and reducing their invasive capacity [83] (Table 4).

On the other hand, C-X-C- chemokine receptor type 4 (CXCR4) plays a crucial role in cancer cell metastasis. The binding of chemokine stromal cell-derived factor 1 (SDF-1 or CXCL12) to the G-protein coupled receptor CXCR4 activates signaling pathways that lead to the expression of genes involved in EMT, contributing to cancer cell migration, invasion, and metastasis. CXCR4 is highly expressed in the TN breast cancer cells MDA-MB-231, where α-mangostin, at a concentration of 10 μM, significantly suppressed cell migration. Additionally, molecular docking simulation suggested a potential interaction between α-mangostin and CXCR4 [88] (Table 4).

#### Inhibition of FAK by α-mangostin in breast cancer

**Fig. 5:**
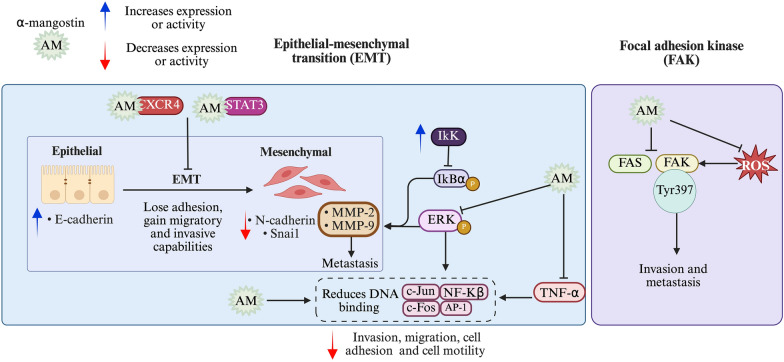
Mechanisms by which α-mangostin inhibits adhesion, invasion and metastasis. The α-mangostin (AM) downregulates mesenchymal markers (N-cadherin, vimentin, Snail) while upregulating the epithelial marker E-cadherin, counteracting EMT progression. AM suppresses TPA-induced expression and activity of matrix metalloproteinases (MMP-2 and MMP-9) by inhibiting ERK phosphorylation and reducing c-Fos, c-Jun DNA binding activity and reducing TNF-α-induced NF-κB and AP-1 nuclear translocation. AM increases IκK expression, blocking IκBα phosphorylation and further decreasing MMP expression. AM also inhibits STAT3 activation, reducing EMT-associated gene expression, and impairs CXCR4 signaling, inhibiting migration and invasion. Disrupts FAK activation by inhibiting phosphorylation. Additionally, AM downregulates FAS, which may attenuate FAK activity. Figure created using BioRender.com

Activation of FAK through phosphorylation at the Tyr^397^ active site is essential for cancer progression and metastasis by modulating these cellular processes. Interestingly, α-mangostin (1–4 µM/24 h) disrupts FAK activation by blocking Tyr^397^ phosphorylation at the active site, thereby reducing its interaction with extracellular matrix-associated proteins in MCF-7 and MDA-MB-231 breast cancer cells. Furthermore, FAK phosphorylation was downregulated following FAS silencing, suggesting that inhibiting FAS may attenuate FAK activity. Thus, the antimetastatic effect of α-mangostin may be associated with its inhibitory action on FAS [75] (Table 4 and Fig. 5).

### Antiangiogenic effects of α-mangostin in breast cancer

Tumor angiogenesis is essential for the growth and progression of solid tumors, and the VEGF/VEGFR axis is a key driver this process. In mammals, the VEGF family includes VEGF-A, VEGF-B, VEGF-C, and placental growth factor (PLGF), with VEGF-A being the most studied and commonly referred to as VEGF. 

The primary receptors for VEGF are VEGFR-1 and VEGFR-2. VEGFR-1 is involved in hematopoiesis, MMPs activation, and the recruitment of monocytes and other immune cells into the tumor microenvironment. VEGFR-2 plays a central role in vasculogenesis and angiogenesis.

**Fig. 6:**
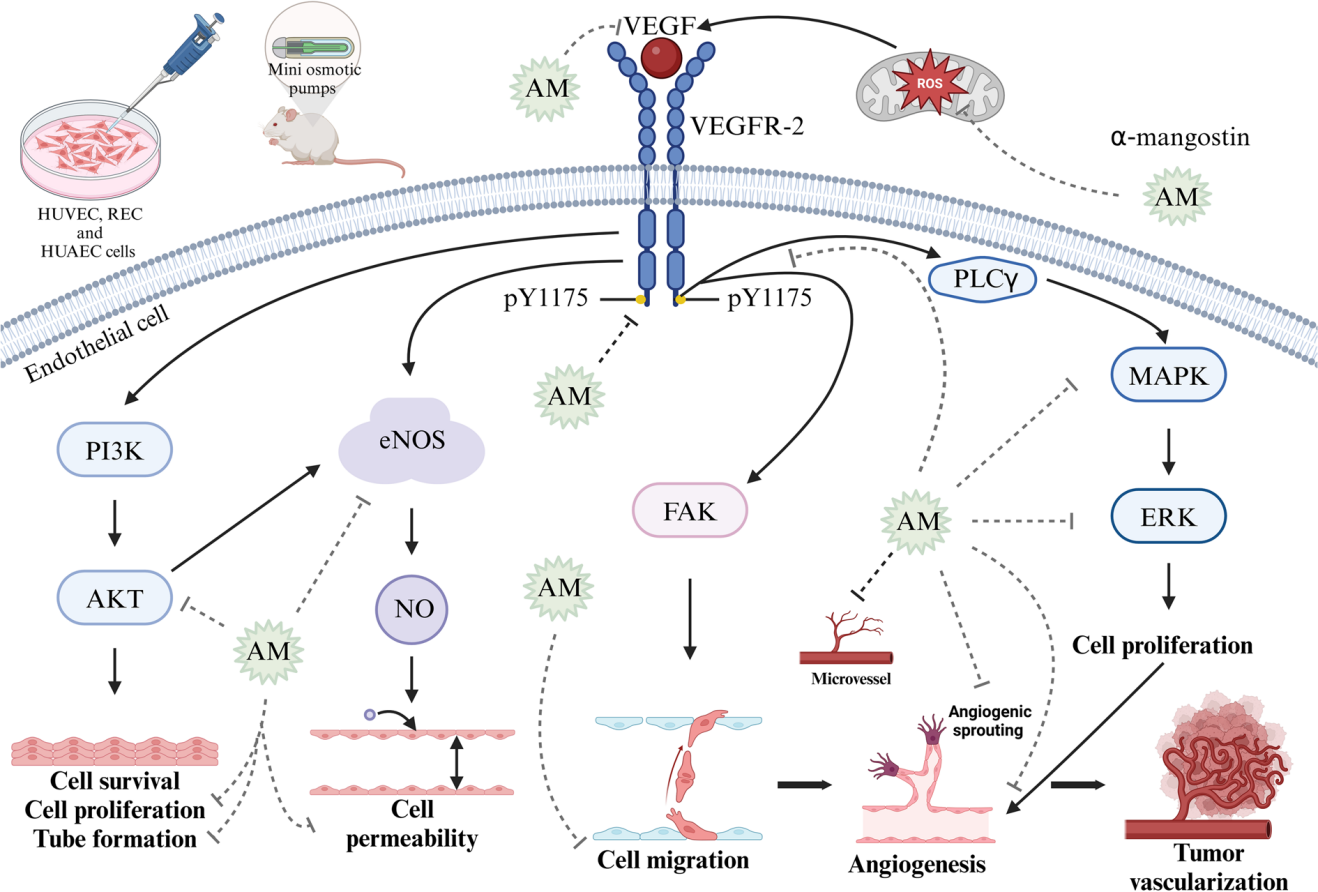
Antiangiogenic effects of α-mangostin in endothelial cells. α-Mangostin (AM) exerts its antiangiogenic effects through multiple mechanisms. AM reduces reactive oxygen species (ROS) production in hypoxic endothelial cells and decreases VEGF protein expression. It also inhibits VEGFR-2 phosphorylation at tyrosine residue Y1175, thereby disrupting key downstream signaling pathways involved in angiogenesis. These include the inhibition of AKT phosphorylation, inhibiting endothelial cell proliferation, and tube formation. AM reduces vessel permeability by reducing endothelial nitric oxide synthase (eNOS) activity. Also, VEGFR-2 inhibition prevents MAPK/ERK1/2 activation, reducing endothelial cell proliferation. Additionally, AM inhibits angiogenic sprouting, endothelial cell migration, and microvessel density. These effects have been demonstrated in both in vitro models (HUVECs, RECs, and HUAECs) and in vivo murine xenograft models. Figure created using BioRender.com

Binding of VEGF to its receptor VEGFR-2 initiates multiple signaling pathways that play essential roles in vascular function. One key pathway involves nitric oxide synthase (NOS), where endothelial NOS (eNOS) and inducible NOS (iNOS) mediates the release of nitric oxide (NO), a potent vasodilator that increases vessel permeability. Another essential signaling pathway is the PI3K/AKT, where PI3K activation initiates downstream activation of PKB, promoting endothelial cell survival, proliferation, and tube formation. Additionally, activation of FAK pathway induces endothelial cell migration, further promoting angiogenesis [124] (Fig. 6). Specifically, VEGF binding to VEGFR-2 induces phosphorylation at key tyrosine residues, including Y951, Y1054, Y1059, Y1175, and Y1214. Among these, phosphorylation of Y1175 is particularly important as it creates binding sites for phospholipase C gamma (PLCγ). This interaction activates the MAPK/ERK1/2 signaling pathway, conducting endothelial cell proliferation, a critical step in angiogenesis [125].

Interestingly, α-mangostin at 10 μM inhibits VEGFR-2 phosphorylation by targeting the Y1175 residue. This inhibition effectively reduces the proliferation of Human Umbilical Vein Endothelial Cells (HUVEC) and Human Umbilical Artery Endothelial Cells (HUAEC), with IC_50_ values of 1.2 μM and 2.4 μM, respectively. Furthermore, α-mangostin inhibits HUVEC migration with an IC_50_ value of 0.03 μM and suppresses tubule formation at concentrations of 0.6 μM and 1.2 μM (Fig. 6) [121]. ROS production induces VEGF expression in Bovine Retinal Endothelial Cells (REC) under hypoxic conditions. Notably, α-mangostin at concentrations of 1 μM, 4 μM, and 8 μM inhibited concentration-dependent ROS production, attenuated VEGF-induced endothelial cell hyperpermeability, and suppressed VEGF-induced endothelial cell proliferation, migration, and 3D tube formation. Additionally, α-mangostin blocked angiogenic sprouting in the ex vivo aortic ring assay. Additionally, α-mangostin inhibited VEGF-induced phosphorylation of VEGFR-2 and the downstream activation of MAPK/ERK1/2-signalling, which are key pathways in angiogenesis [122]. Moreover, it has been demonstrated that the ethanol pericarp extract of mangosteen, predominantly composed of α-mangostin, can reduce VEGF protein expression in T47D breast cancer cells [123] (Table 4).

An in vivo study demonstrated that panaxanthone, comprising approximately 80% of α-mangostin and 20% of γ-mangostin, exhibits both antitumoral and antiangiogenic effects. Specifically, panaxanthone administered at 5000 ppm in the diet of mice xenografted with BJMC3879 murine mammary cancer cells, significantly reduced microvessel density within mammary tumors, indicating a marked suppression of tumor angiogenesis [85] (Table 4).

Similarly, another study demonstrated that α-mangostin treatment at 10 and 20 mg/kg/day, administered via mini osmotic pumps to mice xenografted with BJMC3879luc2 cells, reduced microvessel density in mammary tumors [78]. The mechanism by which α-mangostin exerts its antiangiogenic effects may involve a significant decrease in phospho-AKT levels at Thr^308^ both in vitro (α-mangostin treatment at 20 μM for 3 and 6 h) and in vivo models, suggesting downstream inhibition of AKT signaling pathways [85]. Once activated, AKT phosphorylates various downstream targets involved in critical cellular processes such as cell proliferation, DNA repair, metabolism, cell cycle progression, cell survival, and angiogenesis. In the context of angiogenesis, AKT activates eNOS through phosphorylation, promoting nitric oxide production and facilitating angiogenesis [126]. The inhibition of AKT phosphorylation by α-mangostin may also suppress eNOS activity, further contributing to its anti-angiogenic effects [78] (Table 4).

### Targeting PD-L1: α-mangostin´s potential in breast cancer immunomodulation

The immune checkpoint Programmed Death-Ligand 1 (PD-L1) plays a crucial role in breast cancer, particularly in the interaction between the immune system and cancer cells. PD-L1 is expressed on the surface of some cancer cells, including those in breast cancer, and binds to the PD-1 receptor on T cells. This interaction deactivates the T cells, preventing them from attacking cancer cells, thus facilitating immune evasion. Besides aiding in immune evasion, PD-L1 also promotes tumor growth and progression through various mechanisms [127]. Given this, therapies targeting the PD-1/PD-L1 pathway show great promise for immunotherapy. Drugs that block PD-L1 or PD-1 can reactivate T cells, allowing them to target cancer cells.

In MDA-MB-231 breast cancer cells, which have high levels of PD-L1, both α-mangostin (10 µM) and the mangosteen pericarp ethanol extract (10 µg/mL) significantly inhibit PD-L1 protein expression when treated for 72 h. In silico analysis shows that α-mangostin binds inside PD-L1 dimer pockets, suggesting that α-mangostin stabilized the dimer form, potentially leading to PD-L1 degradation [86].

### Modulation of non-coding RNAs by α-mangostin in breast cancer

The xanthone α-mangostin, also may exert antitumor activity by modulating non-coding RNAs (ncRNAs), which play essential roles in gene expression and chromatin structure regulation. NcRNAs, transcribed from the non-coding DNA in the human genome, are classified into long non-coding RNAs (lncRNAs) and microRNAs (miRNAs). LncRNAs, over 200 nucleotides in length, regulate transcription, influence RNA splicing and stability, and indirectly modulate miRNA activity by acting as molecular sponges. MiRNAs, about 22 nucleotides long, bind to specific mRNA sequences to modulate gene expression, typically affecting mRNA stability or translation. The expression of ncRNAs and miRNAs is tightly regulated; while their dysregulation is linked to diseases, such as cancer, where they act as oncogenes or tumor suppressors [128].

Although no studies have specifically explored the antitumor role of α-mangostin in breast cancer through the direct modulation of lncRNAs or miRNAs, some research has investigated these effects in other cancer models and precursor lesions. In this context, a recent study investigated the antineoplastic effects of α-mangostin in oral submucous fibrosis, a premalignant condition characterized by chronic inflammation, submucosal fibrosis, and myofibroblast activation. This study demonstrated that α-mangostin reduces myofibroblast viability without affecting normal oral cells and inhibits myofibroblast activation, TGF-β/Smad2 signaling, and fibrosis markers through the suppression of LincROR, a lncRNA overexpressed in fibrosis [129]. This lncRNA-mediated mechanism underlies the protective effect of α-mangostin in preventing the progression of oral submucous fibrosis to oral cancer.

Regarding the relationship between miRNAs and the antitumor effects of α-mangostin in cancer, a study conducted on human pancreatic cancer MIA PaCa-2 cells demonstrated that α-mangostin induces autophagy through an AMPK/mTOR and p38-dependent mechanism. Notably, a miRNA PCR array profiled 84 miRNAs in MIA PaCa-2 cells following treatment with α-mangostin. Most miRNAs were found to be downregulated, such as miR-18a, while two, miR-146a and miR-302c, were up-regulated by more than twofold. TaqMan assays further corroborated the reduced expression of miR-18a [130].

Two additional studies conducted in colon cancer cells have investigated the role of α-mangostin as a significant modulator of miRNA expression. In human colon cancer DLD-1 cells, treatment with α-mangostin reduced cell viability by promoting apoptosis. Furthermore, when combined with conventional chemotherapeutic agents such as 5-fluorouracil, α-mangostin enhanced growth inhibition in these cells. Notably, α-mangostin increased the levels of miR-143, a miRNA known to negatively regulate Erk5, a key member of the MAPK family involved in cell proliferation and survival [131]. In a separate study, Kumazaki et al. explored therapeutic strategies to overcome resistance to tumor necrosis factor-related apoptosis-inducing ligand (TRAIL) in DLD-1 cells and cancer stem-like breast epithelial MCF10A cells. They identified that the expression of death receptor 5 (DR5) is critical for TRAIL sensitivity. Remarkably, α-mangostin reversed TRAIL resistance by upregulating DR5 through the downregulation of miR-133b, a negative regulator of DR5 expression [132].

Overall, these studies underscore the potential significance of miRNAs in the antitumor mechanisms of α-mangostin and emphasize the need for further research in this promising yet underexplored area.

## α-Mangostin as an adjuvant of conventional therapies in breast cancer

In breast cancer, the antineoplastic effects of α-mangostin have been evaluated in combination with doxorubicin, 5-fluorouracil and tamoxifen, modifying its efficacy and metabolism. In MCF-7 cells, the ethanolic extract of mangosteen, combined with doxorubicin, increases the cytotoxic activity of the chemotherapeutic agent, which could be related to the induction of apoptosis in neoplastic cells and the inhibition of P-glycoprotein expression [133]. Another study reported that in spheroids formed by MCF-7 cells, the combination of α-mangostin and doxorubicin resulted in significant cytotoxicity and notable inhibition of retinaldehyde-dependent aldehyde dehydrogenase (RALDH) isoenzyme activity, which suggests that this combination could be effective in reducing cell stemness [2]. Furthermore, it has been determined that α-mangostin synergizes the antineoplastic effect of the chemotherapeutic agent 5-fluorouracil in breast cancer cells with different phenotypes, allowing for a dose reduction [3]. Additionally, in MCF-7 and T-47D breast cancer cells, the combination of α-mangostin with 4-hydroxy-tamoxifen not only promoted a synergistic antiproliferative effect but also allowed for a dose-reduction of both compounds. Moreover, this combination inhibited, to a greater extent, the gene expression of *KCNH1*, *CCND1*, and *BIRC5* (survivin), markers of oncogenesis, cell cycle progression, and inhibition of apoptosis, respectively [4].

## Antitumor effects of α-mangostin in breast cancer murine models

Panaxanthone inhibited tumor growth and metastasis in a mouse model of breast cancer. BJMC3879 cells, a mammary adenocarcinoma cell line highly metastatic to the lungs and lymph nodes with p53 mutation, were subcutaneously (s.c.) inoculated into mice, generating tumors two weeks later. Treatment began at 8 weeks with 2500 ppm and 5000 ppm of panaxanthone. At 2500 ppm, tumor growth and lung metastasis multiplicity were inhibited, with a greater effect observed at 5000 ppm. The antitumor effects of panaxanthone were associated with increased apoptosis, inhibition of cell proliferation (inhibition of PCNA), and anti-angiogenesis (reduced microvasculature density) [85].

In another mouse model study, BJMC3879luc2 cells were s.c. xenografted (2.5 million/0.3 mL of PBS). Three weeks later, with tumor diameters between 0.4 and 0.6 cm, mice were treated with 0, 10 or 20 mg/kg/day of α-mangostin via mini osmotic pumps for 6 weeks. Αt a dose of 20 mg/kg, α-mangostin decreased tumor volume and metastasis to lymph nodes, increased apoptotic cell death, elevated expression of active caspase-3 and -9, and reduced microvasculature density, cell migration via lymphatic vessels, and phosphorylation of AKT [78].

It is widely acknowledged that chronic inflammation, induced by factors such as infections, obesity, and genetic mutations, promotes immunosuppression and contributes to cancer progression as well as aging-related metabolic disorders [134]. A study found that α-mangostin reduces pro-inflammatory cytokines and chemokines in mice, alleviating adiposity, hyperlipidemia, and insulin resistance. It also reduced macrophage content and shifted their polarization, protecting against liver injury by suppressing miRNA-155-5p secretion, making α-mangostin a promising candidate for treating inflammation and aging-related metabolic disorders [135].

In a study using the rat LA7 mammary adenocarcinoma cells model, cells were injected into the mammary fat pads of Sprague–Dawley rats (2.0 million/0.1 mL). Ten days post-implantation, the rats were treated orally via gastric tube with 30 and 60 mg/kg of α-mangostin (from *Cratoxylum arborescens*) dissolved in Tween 20 and s.c. with 10 mg/kg of tamoxifen dissolved in the same vehicle. Tumors in the control group grew rapidly, reaching an average volume of 1737 ± 563 mm^3^ by day 28. In contrast, groups treated with 30 mg/kg and 60 mg/kg of α-mangostin showed a significant reduction in tumor volume compared to the control group, with 60 mg/kg of α-mangostin resulting in a greater reduction. Specifically, α-mangostin at 30 mg/kg reduced tumor volume by 74.1%, while the highest inhibition of 79.2% was observed with 60 mg/kg. For comparison, tamoxifen at 10 mg/kg decreased tumor volume by 83.6% [119].

Using the same rat breast cancer model, it was found that α-mangostin not only protects the rat mammary gland from the tumorigenic effects of LA7 cells by increasing apoptosis and suppressing proliferation (as shown by reduced PCNA and increased p53 expression), but also induces antioxidant effects and lowers the serum levels of the cancer biomarkers Carcinoembryonic Antigen and Cancer Antigen 15–3 [136].

## Clinical trials involving mangosteen

To present date, no clinical trials investigating mangosteen products as anticancer agents have been conducted. Instead, most clinical trials have focused on evaluating their anti-oxidant, anti-inflammatory and anti-obesity effects, as well as their impact on neurological disorders, safety, and tolerability [137–142].

### Safety, bioavailability, antioxidant and anti-inflammatory effects of mangosteen

Few studies have been developed to determine the absorption, bioavailability, antioxidant, and anti-inflammatory effects of mangosteen product consumption, as shown in Table 5.

**Table 5:** In vivo studies of bioavailability and antioxidant effects of mangosteen beverages

Beverage or product	Aim	Amount intake	Design of study	Findings	Ref
Pure mangosteen juice containing 5.3 ± 0.1 mmol/L of total xanthones, with α-mangostin, garcinones C, D, and E, γ-mangostin, gartanins, and other identified xanthones comprising 58%, 2%, 6%, 4%, and 5% of the total, respectively	Determine the bioavailability of xanthones from pure mangosteen juice	Single dose of 60 mL with a high-fat breakfast	10 healthy adult participants consumed pure mangosteen juice with a high–fat breakfast	α-Mangostin was the only xanthone detected in serum, with Cmax values ranging from 42 to 450 nmol/L. The Tmax for total α-mangostin was 2–4 h in 8 participants and 8 h in the remaining 2 participants. Xanthone levels in 24 h urine samples ranged from 0.9 to 11.1 µmol/L, accounting for 2.0 ± 0.3% of the ingested dose	[143]
Mangosteen-based functional beverage containing mangosteen, aloe vera, green tea, and vitamins A, C, D_3_, E, and B complex	Determine the bioavailability and antioxidant effects	Single dose of 59 mL before breakfast	A 24 h randomized, double-blind, placebo-controlled clinical trial was conducted with 20 healthy young adults (10 men and 10 women, aged 20 to 23 years old). Participants were randomly assigned to receive a functional beverage or a placebo	The Cmax of α-mangostin was 3.12 ± 1.47 ng/mL, reaching a Tmax of 1 h after consumption. The concentration decreased to one-third of Cmax by the fourth hour and remained stable until the sixth hour. Additionally, antioxidant capacity, measured by the ORAC assay, increased by over 16% at 1 h after consumption, with ORAC values rising by up to 18% after 2 h and remaining elevated for 6 h	[144]
Mangosteen-based functional beverage containing mangosteen, aloe vera, green tea, and multivitamins	Determine de bioavailability and antioxidant effects	Single dose of 245 mL	A 6 h randomized, double-blind, placebo-controlled clinical trial was conducted with 20 healthy adults (10 men and 10 women, aged 18 to 60 years old). Participants were randomly assigned to receive a functional beverage or a placebo	The Cmax of α-mangostin was 4.16 ± 2.85 ng/mL at Tmax of 1 h after consumption; the elevated level of α-mangostin was sustained through the rest of the course of the trial. Furthermore, antioxidant capacity increased as much as 60% at 1 h after consumption, and the antioxidant level lasted at least 6 h	[145]
Mangosteen-based functional beverage containing mangosteen, aloe vera, green tea, and multivitamins	Determine the antioxidant, anti-inflammatory and immune biomarkers	Daily dose consumption of 245 mL	A 30-days randomized, double-blind, placebo-controlled clinical trial was conducted with 60 healthy adults (30 men and 30 women, aged 18 to 60 years old). Participants were randomly assigned to receive a functional beverage or a placebo	Daily consumption of mangosteen-based drink improved antioxidant and anti-inflammatory biomarkers in healthy adults without adverse effects on immune, liver, and kidney functions. The group that received the mangosteen drink showed a 15% increase in antioxidant capacity and a significant 46% reduction in C-reactive protein levels compared to the placebo group. Immunity biomarkers remained unchanged in both groups	[146]
Commercial mangosteen juice	Evaluate the effect of multiple dosages of mangosteen juice (3, 6 or 9 oz twice daily) on indicators of inflammation and antioxidant levels	Consumption of 18 oz ( ~ 532 mL) of mangosteen juice *per* day during 8 weeks	An 8-week randomized; double-blind, placebo-controlled clinical trial was conducted with obese patients who had elevated CRP levels. Participants were randomly assigned to receive three different doses of mangosteen juice or a placebo	The consumption of mangosteen juice reduced CRP levels in obese subjects compared to placebo for those taking 18 oz daily	[147]

*Cmax* maximum plasma concentration, *Tmax* time to reach maximum concentration, *ORAC* oxygen radical absorbance capacity, *CRP* C-reactive protein

An additional clinical trial determined that the oral consumption of mangosteen pericarp polar extract capsules (220–280 mg/day) by healthy subjects is safe for up to 24 weeks without liver or kidney dysfunction and exhibited antioxidant effects [148]. In a clinical trial involving patients with acute exacerbation of chronic obstructive pulmonary disease, the addition of mangosteen pericarp extract (1100 mg/twice a day) during hospitalization was safe and significantly decreased the plasma levels of interleukin (IL)-6 and malondialdehyde, shortening the length of stay [149].

## Adverse effects of α-mangostin both in vitro and in vivo

Regarding the antiproliferative effects of α-mangostin in non-neoplastic cell lines, it was demonstrated that the xanthone exhibits cytotoxicity against the monkey kidney tissue-derived Vero cell line, with a half-maximal cytotoxic concentration (CC_50_) of 7.5 ± 0.04 µM [19]. Another study also found that α-mangostin inhibited Vero cell proliferation with an IC_50_ value of 14.26 µM [74].

Interestingly, a study demonstrated that treating isolated rat platelets with α-mangostin (1–10 μM) inhibited platelet aggregation in a concentration-dependent manner and induced platelet shape changes; while at higher concentrations (25 and 50 µM), α-mangostin caused platelet lysis [150].

Unfortunately, in vitro studies have shown that α-mangostin exhibits hemolytic activity in rabbit red blood cells (red blood cells volume/volume of α-mangostin at 4%) and human blood (α-mangostin at 100 µg/mL, equivalent to 242.62 µM), as indicated by the amount of hemoglobin released from erythrocytes [151, 152]. While this effect has only been observed in vitro, it is mitigated by encapsulating α-mangostin into nanoparticles [153]. To our knowledge, such an effect has not been reported with in vivo administration.

On the contrary, some in vivo studies did not report adverse effects. For instance, the consumption of a diet containing 845 mg/kg of α-mangostin showed no adverse effects in mice [154]. However, a pharmacokinetic study in ICR mice determined that the lethal dose (LD_50_) for intraperitoneal administration of α-mangostin is 150 mg/kg and 231 mg/kg for mangosteen extract [155]. In a murine model of colitis induced by dextran sulfate sodium administration to C57BL/6 J mice, dietary administration of α-mangostin (900 mg of α-mangostin *per* kg of diet) exacerbated the condition. The treated mice exhibited greater colonic inflammation and injury in the colon compared to the control group, along with intestinal dysbiosis and other signs [156]. In general, in clinical trials no side effects have been reported; however, in a case report described in 2008, a 58-years-old patient showed severe lactic acidosis associated with mangosteen juice consumption during the last 12 months as a dietary supplement to lose weight. The medical history of the patient included pulmonary sarcoidosis, metabolic syndrome, and chronic kidney disease [157].

It is important to note that all signs and symptoms of adverse effects depend on the concentration or dose of α-mangostin, as well as on the administration route.

## Conclusion

α-Mangostin, an accessible natural antineoplastic compound derived from the mangosteen tree, represents a promising treatment option for breast cancer, whether used independently or alongside conventional therapy, as supported by numerous preclinical studies. The variety of mechanisms of action associated with α-mangostin makes it a powerful therapeutic alternative; however, much remains to be studied, including its in vivo effects in carefully-designed randomized controlled trials. These trials are urgently needed, as none have been conducted on breast cancer so far. Besides helping to determine α-mangostin therapeutic efficacy, clinical trials will outline the adverse effects derived from α-mangostin administration, allowing to define the adequate dose and administration route.

## Supplementary Information

Supplementary material 1.

## Data Availability

Not applicable.
