# Supplementary material for: Exploring the antineoplastic potential of α-mangostin in breast cancer
Source: Nat Prod Bioprospect. 2025 Jul 3;15(1):43. doi: 10.1007/s13659-025-00528-5 (PMC12229438; doi:10.1007/s13659-025-00528-5)
Supplement: Supplementary file 1 — Supplementary material 1. [file 13659_2025_528_MOESM1_ESM.doc]

**Supplementary table 1 Mangosteen supplements**

| **Brand** | **Name** | **Presentation** | **Content** | **Source** |
| --- | --- | --- | --- | --- |
| **MX3** | Mangosteen xanthone | Box with 60 capsules | Each capsule contains 500 mg of mangosteen fruit pulp powder | [*https://www.mx3ph.com/shop/mx3-capsule*](https://www.mx3ph.com/shop/mx3-capsule) |
| **Savesta** | Superior Antioxidant Mangosteen | Bottle with 60 vegetarian capsules | Each capsule contains 500 mg of mangosteen pericarp extract, with 40% flavonoids (200 mg), 15% α-mangostin (75 mg), 15% tannins (75 mg) | [*https://allnatural.mx/producto/savesta-mangostan-60-capsulas-vegetarianas/*](https://allnatural.mx/producto/savesta-mangostan-60-capsulas-vegetarianas/) |
| **Solaray** | Vital Extracts Mangosteen | Bottle with 60 vegetarian capsules | Each capsule contains 500 mg of mangosteen fruit extract, guaranteed to provide 1750 ORAC units, 75 mg xanthones and 100 mg polyphenols | [*https://solaray.com/products/mangosteen-fruit-extract*](https://solaray.com/products/mangosteen-fruit-extract) |
| **Emerald** | Doctor-Formulated Mangosteen pericarp extract 10:1 | Bottle with 60 vegetarian capsules | Each capsule contains 600 mg of a blend of mangosteen pericarp powder and mangosteen pericarp extract (10:1) | [*https://store.emeraldlabs.com/products/mangosteen-2*](https://store.emeraldlabs.com/products/mangosteen-2) |
| **Advance Physician Formulas** | Mangosteen | Bottle with 60 vegetarian capsules | Each capsule contains 500 mg of mangosteen pericarp extract, standardized to 10% mangostin | [*https://physicianformulas.com/products/mangosteen-500mg-60-capsules*](https://physicianformulas.com/products/mangosteen-500mg-60-capsules) |
| **Biotech Nutritions** | Pure Mangosteen | Bottle with 90 capsules | Each capsule contains 500 mg of mangosteen pericarp-hull powder | [*https://www.biotechnutritions.com/Mangosteen*](https://www.biotechnutritions.com/Mangosteen) |
| **Swanson** | Mangosteen Antioxidant Support | Bottle with 90 capsules | Each capsule contains 500 mg of mangosteen pericarp extract, with 20% polyphenols (100mg) and 10% alpha-mangostin (50 mg) | [*https://www.swansonvitamins.com/p/swanson-superior-herbs-mangosteen-standardized-500-mg-90-caps*](https://www.swansonvitamins.com/p/swanson-superior-herbs-mangosteen-standardized-500-mg-90-caps) |
| **Solaray** | True Herbs Mangosteen | Bottle with 100 vegetarian capsules | Each capsule contains 475 mg of mangosteen whole fruit | [*https://solaray.com/products/mangosteen-fruit*](https://solaray.com/products/mangosteen-fruit) |
| **Swanson** | Full Spectrum Mangosteen | Bottle with 100 capsules | Each capsule contains 500 mg of mangosteen pericarp | [*https://www.swansonvitamins.com/p/swanson-premium-mangosteen-500-mg-100-caps*](https://www.swansonvitamins.com/p/swanson-premium-mangosteen-500-mg-100-caps) |
| **Healthy Superfoods** | Mangostan | Bottle with 100 capsules | Each capsule contains 500 mg of mangosteen fruit | [*https://www.healthysuperfoods.mx/healthy-superfoods-mangostanmangosteen-organico-premium-100-capsulas-500mg-sabor-natural/p/MLM26024089*](https://www.healthysuperfoods.mx/healthy-superfoods-mangostanmangosteen-organico-premium-100-capsulas-500mg-sabor-natural/p/MLM26024089) |
| **Source Naturals** | Mangosteen antioxidant and immune support | Bottle with 30, 60 or 120 tablets | Each tablet contains 187.5 mg of mangosteen pericarp extract, standardized to 90% of flavonoids and 40% mangostins, providing 75 mg of xanthones as mangostins | [*https://www.sourcenaturals.com/products/GP1846*](https://www.sourcenaturals.com/products/GP1846) |
| **GoNutra** | Mangosteen | Bag with 1lb of mangosteen powder | Mangosteen fruit powder | [*https://gonutra.com/products/mangosteen-powder-pericarp-1lb-superfood*](https://gonutra.com/products/mangosteen-powder-pericarp-1lb-superfood) |
| **Healthy Superfoods** | Mangosteen | Bag with 500 g of mangosteen powder | Mangosteen fruit powder, bag with 500 g | *[https://www.healthysuperfoods.mx/MLM-2893178430-mangostan-organico-500g-envio-gratis-_JM#position%3D4%26search_layout%3Dgrid%26type%3Ditem%26tracking_id%3Db1b325c6-f31c-4a1a-b8dd-3a2d1efa309a](https://www.healthysuperfoods.mx/MLM-2893178430-mangostan-organico-500g-envio-gratis-_JM#position%3D4%26search_layout%3Dgrid%26type%3Ditem%26tracking_id%3Db1b325c6-f31c-4a1a-b8dd-3a2d1efa309a)* |
| **Swanson** | Mangosteen concentrate | Bottle of juice 32 fl oz (946 mL) | Mangosteen fruit puree, pineapple juice concentrate, white grape juice concentrate, concord grape juice concentrate, mangosteen fruit powder, red raspberry juice concentrate, strawberry juice concentrate, xanthan gum, cranberry juice concentrate, citric acid, blueberry juice concentrate, dark sweet cherry juice concentrate, and mangosteen pericarp powder. | [*https://www.swansonvitamins.com/p/swanson-ultra-mangosteen-liquid-32-fl-oz-946-ml-liquid*](https://www.swansonvitamins.com/p/swanson-ultra-mangosteen-liquid-32-fl-oz-946-ml-liquid) |
| **Dynamic Health** | Mangosteen Gold | Bottle of juice 16 (473 mL) or 32 fl oz (946 mL) | Each 30 mL serving contains 16 g of mangosteen fruit | [*https://dynamichealth.com/products/mangosteen-gold*](https://dynamichealth.com/products/mangosteen-gold) |
| **Isagenix** | Xango mangosteen beverage/Xango Reserve mangosteen beverage | Bottle of juice 25.36 fl oz (750 mL) | Whole mangosteen puree, along with grape juice concentrate, pear juice concentrate, cranberry juice concentrate, and cherry juice concentrate | [*https://www.isagenix.com/es-us/shop/daily-nutrition/xango?pid=052036640a854c6294cd1c505d48a467*](https://www.isagenix.com/es-us/shop/daily-nutrition/xango?pid=052036640a854c6294cd1c505d48a467) |
| **Phytovit** | Mangostan C | Box with 20 drinkable vials of 15 mL | Each vial contains 6 g of mangosteen puree fruit, 100 mg of mangosteen dry extract (10:1), 50 mg of Myrciaria dubia dry extract (providing 50% of vitamin C), and 50 mg of blue berry dry extract (containing 25% pronthocyanidins) | [*https://www.phytovit.com/inicio/articulo/mangostan*](https://www.phytovit.com/inicio/articulo/mangostan) |
| **Swanson** | Full Spectrum Super Fruit Complex with mangosteen, pomegranate and noni | Bottle with 90 capsules | Each capsule contains 240 mg of mangosteen fruit, 240 mg of noni fruit, and 240 mg of pomegranate. | [*https://www.swansonvitamins.com/p/swanson-premium-mangosteen-pomegranate-noni-complex-240-240-240-mg-90-caps*](https://www.swansonvitamins.com/p/swanson-premium-mangosteen-pomegranate-noni-complex-240-240-240-mg-90-caps) |
| **VIP Vitamins** | Antioxidant Mega Complex | Bottle with 60 capsules | Each capsule contains 150 mg of acai fruit extract, 150 mg of goji berry fruit extract, 150 mg of noni fruit extract, 125 mg of mangosteen fruit extract, 125 mg of pomegranate extract, and 10 mg of Polygonum Cuspidatum extract as trans-resveratrol | [*https://www.vipvitamins.com/product-page/antioxidant-mega-complex*](https://www.vipvitamins.com/product-page/antioxidant-mega-complex) |

Access date: January 21, 2025
